# Language Models Enable Data-Augmented Synthesis Planning for Inorganic Materials

**DOI:** 10.1021/acsami.5c11229

**Published:** 2025-11-26

**Authors:** Thorben Prein, Elton Pan, Janik Jehkul, Steffen Weinmann, Elsa Olivetti, Jennifer L. M. Rupp

**Affiliations:** † School of Natural Sciences, Technische Universität München, Garching bei München 85748, Germany; ‡ Munich Data Science Institute, Garching bei München 85748, Germany; § TUMint. Energy Research GmbH, Garching bei München 85748, Germany; ∥ Department of Materials Science and Engineering, 2167 Massachusetts Institute of Technology, Cambridge, Massachusetts 02139, United States; ⊥ School of Computation, Information and Technology, Technische Universität München, Garching bei München 85748, Germany

**Keywords:** large language models, solid-state synthesis, precursor recommendation, synthesis condition prediction, synthetic data augmentation

## Abstract

Inorganic synthesis planning currently relies primarily on heuristic approaches or machine learning models trained on limited data sets, which constrains its generality. We demonstrate that language models (LMs) without task-specific fine-tuning can recall synthesis conditions reported in the scientific literature. Off-the-shelf models, such as GPT-4.1, Gemini 2.0 Flash, and Llama 4 Maverick achieve a Top-1 precursor prediction accuracy of up to 53.8% and a Top-5 performance of 66.8% on a held-out set of 1000 reactions. They also predict calcination and sintering temperatures with mean absolute errors of <126 °C, matching or surpassing specialized regression models. Ensembling these LMs further enhances predictive accuracy and reduces inference cost per prediction by up to 70%. Given the broad, cross-domain knowledge of LMs, we evaluate whether they enable knowledge transfer by training a transformer, SyntMTE, on 28,548 LM-generated reaction recipes. Compared to a model trained on literature-reported data, we find that a model trained solely on LM-generated data exhibits competitive performance (only 6% worse). Conversely, a model trained on both the LM-generated and literature-reported data improves performance by up to 4%. In a case study on Li_7_La_3_Zr_2_O_12_ solid-state electrolytes, we demonstrate that SyntMTE reproduces the experimentally observed dopant-dependent sintering trends. Our hybrid workflow enables scalable and data-efficient inorganic synthesis planning.

## Graphical abstract

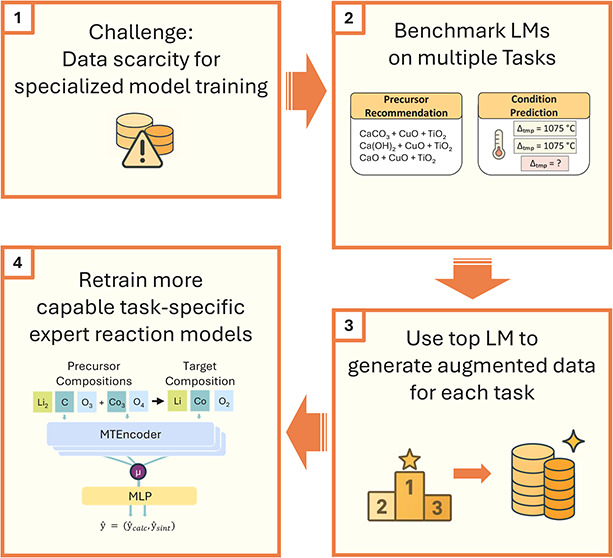

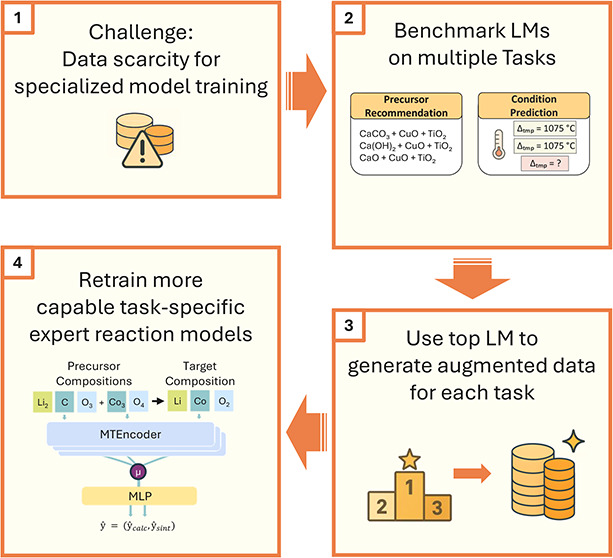

## 1 Introduction

The discovery and design of advanced materials underpin progress in energy conversion and storage, information technology, and medicine.^1–5^ Recent advances in machine learning (ML) accelerated simulations have driven a rapid increase in candidate materials computationally predicted, now numbering in the millions.^6,7^ As a result, the synthesis of these candidates has become the principal bottleneck in the materials discovery pipeline.^8–12^ Although density functional theory (DFT) provides valuable thermodynamic insight, it remains challenging to accurately predict kinetics, diffusion, or phase-transformation pathways, leaving synthesis largely a trial-and-error process.^9,13–16^

Accordingly, researchers have adopted ML methods to extract synthesis protocols from the scientific literature and predict feasible reaction pathways for novel structures.^17–19,90^ Foundational studies by Kononova et al.,^16^ Kim et al.,^20^ and Huo et al.^21^ curated comprehensive synthesis databases, thereby enabling ML-based approaches to inorganic synthesis planning. Recent efforts have focused on ML methods for two tasks: (i) precursor recommendation, i.e., identifying suitable reagent combinations; and (ii) synthesis-condition prediction, i.e., determining optimal reaction parameters. These tasks are then applied sequentially to propose a viable synthesis protocol for a target material.

### 1.1 Precursor Recommendation

Most precursor recommendation methods focus on solid-state synthesis. An example of LiCoO_2_ is illustrated in Table 1, where a model ranks suitable precursor combinations by their likelihood. Kim et al.^22^ used an RNN with ELMo embeddings to extract over 50,000 synthesis actions and 116,000 precursor mentions and subsequently trained a paired conditional VAE to jointly model action sequences and precursor formulas, enabling plausible precursor suggestions for novel targets. Kim^23^ introduced an element-wise retrosynthesis with 39 template classes, while He et al.^24^ developed a retrieval-based model using attention to compare planned synthesis to historical routes, extended by Noh et al.^25^ via an enthalpy-aware ranker. Prein et al.^26^ improved generalization by embedding materials with a pretrained transformer using a pairwise ranker to assess precursor suitability for unseen compounds.

**Table 1:**
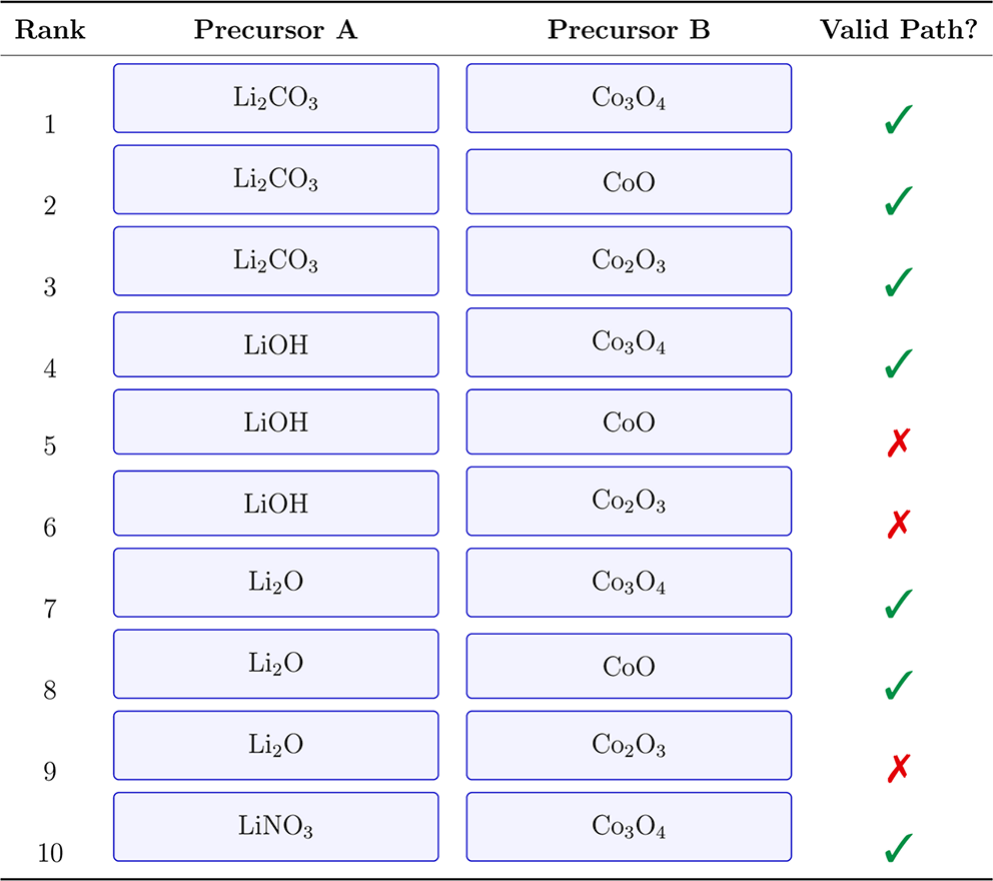
Top-10 LiCoO_2_ Precursor Sets^a^

a Predictions from GPT-4.1 assuming a standard solid-state synthesis protocol.

### 1.2 Synthesis Condition Prediction

After precursor selection, a second model predicts the isothermal-hold temperatures and dwell times for both the calcination and the sintering steps in solid-state synthesis routes. Huo et al.^27^ applied linear and tree-based regression on text-mined features (e.g., melting points and formation energies), achieving a mean absolute error (MAE) of approximately 140 °C. Prein et al.^28^ developed a Reaction Graph Network using MTEncoder embeddings and graph-attention layers. Pan et al.^29,30^ framed condition prediction as a diffusion-based generative task conditioned on the target material’s structure, capturing the one-to-many nature of structure-synthesis relationships.

Despite the preponderance of ML methods, models are still bottlenecked by data-centric concerns. Synthesis databases remain relatively small, only rarely exceeding a few × 10^3^ unique entries, leaving the majority of chemistries unrepresented (Figure 4).^9,18^ The limited size of existing data sets inhibits recovery of the true distribution of processing parameters for most materials, thereby precluding the robust mapping of a “synthesis window”, the region of temperature and isothermal dwell-time combinations that yield the desired phase. In spirit, this mapping parallels long-standing tools in experimental synthesis, most notably, phase-stability regimes in classical time-temperature transformation (TTT) diagrams.^31,32^ Automated text-mining pipelines further degrade data quality by introducing extraction errors, such as misassigned stoichiometries, omitted precursor references, and conflation of precursor and target species, particularly in complex multistep protocols. Consequently, ML models trained on these sparse, noisy data sets cannot confidently resolve the underlying “synthesis window”, leading to diminished predictive accuracy and poor generalization to novel materials.

**Figure 1:**
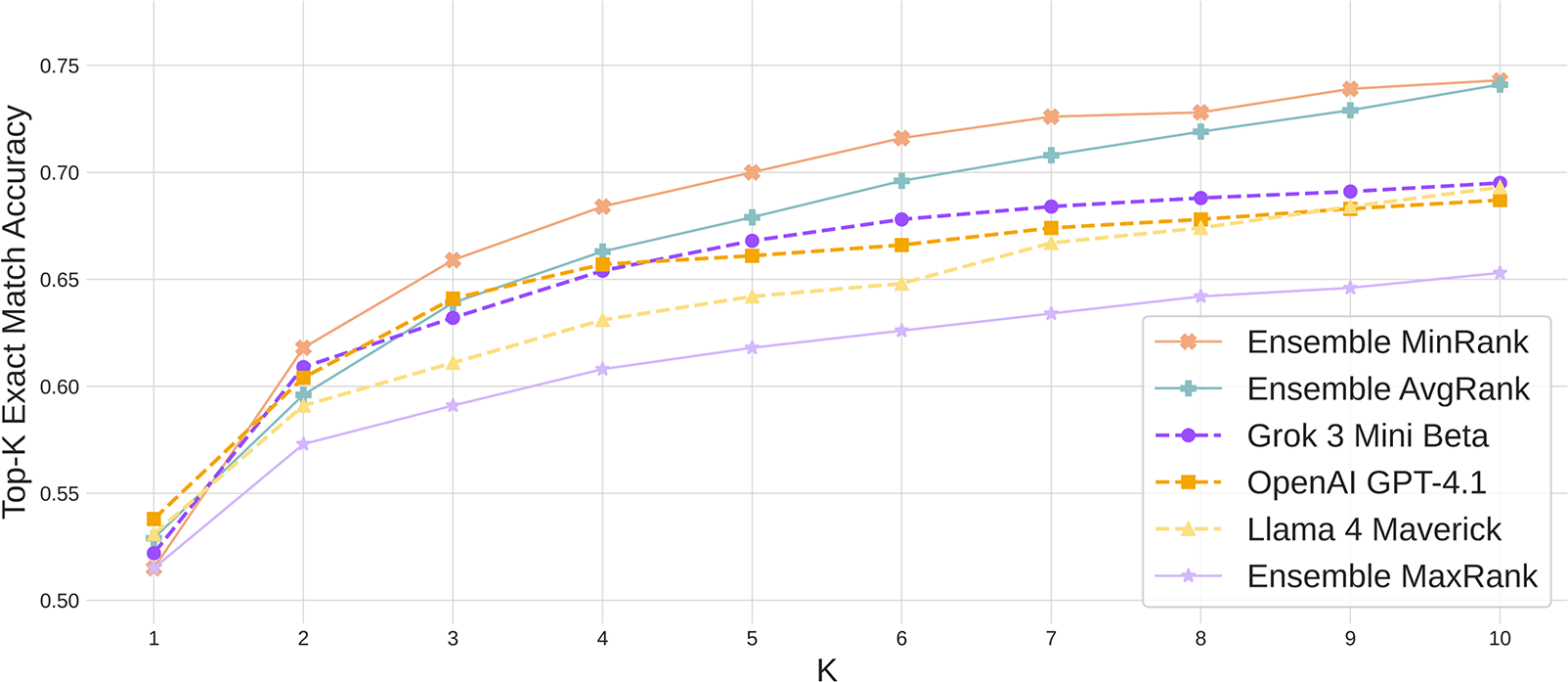
Ensemble comparison. Top-*k* exact match accuracy for three individual LMs: Grok 3 mini Beta, GPT-4.1, and Llama 4 Maverick and their joint ensemble with predictions combined using minimum-rank, average-rank, and maximum-rank voting. The minimum-rank ensemble achieves the best recall beyond Top-1.

### 1.3 Language Models in Materials Synthesis

In contrast, pretrained LMs are trained on orders of magnitude more unstructured chemical knowledge: implicit heuristics, phase-diagram insights, and procedural narratives from their extensive pretraining corpora.^80–89^ They have demonstrated considerable utility across scientific disciplines.^33–37^ Generative LMs have excelled in crystal-structure generation: CrystaLLM^38^ and Crystal-Text-LLM^39^ produce DFT-validated geometries, while FlowLLM^40^ refines LLM-generated structures using flow matching.^41^ In synthesis planning, GPT variants fine-tuned for synthesizability and precursor prediction may match specialized models.^42^ ALDbench evaluates LMs on open-ended atomic layer deposition questions and finds that GPT-4o scores a reasonable 3.7 out of 5, resembling a passing grade.^43^ However, current state-of-the art LMs have never been systematically evaluated on generating precursors and processing conditions for solid-state inorganic synthesis, the crucial domain for materials discovery.^12^ Benchmarking LMs in this domain allows researchers to make informed decisions on LM performance, as well as guide their choice toward certain LMs. Beyond establishing a benchmark featuring current state-of-the-art models, we investigate directions of immediate relevance to materials synthesis using ML. We compare LM ensembles with single-model workflows and evaluate generated synthesis condition distributions. We probe whether LM-generated data can augment literature-mined data, i.e., whether synthetic data can serve as a prior for domain-specific models. To the best of our knowledge, these research directions have not been systematically explored in prior work. This work aims to answer the following questions:

1. How well do state-of-the-art LMs perform on inorganic solid-state synthesis planning tasks?
2. Do LM ensembles outperform single models, and how do they alter the distribution of proposed recipes?
3. Can LM-generated synthesis recipes enrich current literature-mined sparse databases and act as an informative prior for domain-specific models?

We find that synthetic LM-generated data boosts the prediction accuracy. We leverage the models trained on the augmented data set in a case study on doped Li_7_La_3_Zr_2_O_12_ (LLZO) compounds, a functional ceramic whose cubic phase is challenging to stabilize and therefore requires careful selection of dopants and sintering steps.^44–46^ Overall, our contributions are as follows:

- We demonstrate strong performance of LMs in recalling previously reported synthesis trends. Moreover, we demonstrate that an ensemble of LMs surpasses individual models in precursor suggestion and processing-condition prediction, and can even accurately reconstruct synthesis condition distributions from the literature.
- We show how LM-generated data can be beneficial to domain-specific expert models by curating a synthetic data set of 28,548 complete LM-generated solid-state synthesis recipes, which signifies a 6-fold increase in complete entries over existing solid-state synthesis data sets.^16^
- Leveraging both literature-mined and synthetic data, we develop a transformer-based model (SyntMTE) to regress synthesis conditions for solid-state synthesis. This model outperforms previous methods , such as CrabNet, composition-based neural networks, and decision-tree regressors, while models exhibit significant improvements up to 4% when initially trained on the synthetic data set.

## 2 Results and Discussion

### 2.1 Benchmarking LMs in Inorganic Materials Synthesis

To assess LM capabilities in inorganic synthesis planning, we deploy state-of-the-art LMs in two solid-state synthesis tasks. We use a data set derived from Kononova et al.,^16^ which contains roughly 10,000 unique precursor–target material combinations. For precursor recommendation, we randomly sample a 1000 data point test data set in accordance with previous work.^26^ We submitted prompts to an LM provider (via OpenRouter) without specifying the number of precursors, thereby requiring each model to infer the appropriate precursor count for each reaction (Listing A1). For synthesis condition prediction, a 1000 entry data set is sampled by filtering for recipes containing complete sintering and calcination temperature information. For both tasks, we provide the LMs with 40 in-context examples from the held-out validation fraction of the data set (Figure S1). For precursor prediction, we evaluate the exact match accuracy.^25^ We compare 7 state-of-the-art LMs to cover a diverse set of models. Further details on the different LMs can be found in Supporting Information.

#### 2.1.1 Precursor Recommendation

We evaluate the LMs on the precursor prediction task and report Top-*k* exact match accuracies. The exact match accuracy can be considered a lower bound on performance, as the model is required to reproduce precisely the precursor set reported in the literature, whereas alternative unreported synthesis routes may also exist. Because practical experimental synthesis requires a diverse set of multiple candidate precursor sets, Top-5 and Top-10 metrics are particularly informative, indicating whether a correct precursor set appears within the model’s top 5 or 10 suggestions. Table 2 shows that all LMs deliver competitive performance and cluster within a narrow performance band. Only Qwen 2.5 VL scores lower by a margin. OpenAI GPT-4.1 leads the Top-1 ranking at 53.8% and retains good performance for high *k*. The model is followed by Grok 3 mini Beta and Llama 4 Maverick as well as DeepSeek Chat v3. We compare the LM results with previously published domain-specific ML models from the literature (Table S4). It is notable how the top ranked LMs outperform the domain-specific expert models by a distinct margin. However, the comparison is only partly valid. Baseline models were trained on limited data sets, while LMs may have benefited from data leakage during pretraining on test-set synthesis protocols. The best baseline reported in the literature, Synthesis Similarity,^24^ achieves Top-5 and Top-10 accuracies of 58% and 61%, respectively, while individual LMs attain scores up to 67% and 70%. This example provides a compelling demonstration that state-of-the-art LMs, without any chemistry-specific training objectives, are able to recall high-quality chemistry knowledge through in-context learning only.

**Table 2:** Precursor Recommendation Performance^a^

model	Top-1 ↑	Top-3 ↑	Top-5 ↑	Top-10 ↑
ensemble min-rank 1^b^	52.3	**65.8**	**70.7**	**74.3**
ensemble min-rank 2^b^	51.8	63.1	67.4	71.9
				
OpenAI GPT-4.1	**53.8**	**64.1**	66.1	68.7
Grok 3 mini Beta	52.2	63.2	**66.8**	**69.5**
Llama 4 Maverick	53.1	61.1	64.2	69.3
DeepSeek Chat v3	53.5	60.7	63.7	66.2
Mistral Small 3.1	52.0	59.7	61.7	63.9
Gemini 2.0 Flash	51.4	59.2	62.0	66.2
Qwen 2.5 VL	50.7	55.5	58.0	59.3

a Top-*k* exact-match accuracies for individual LMs and ensemble strategies on retrosynthesis precursor prediction. GPT-4.1 achieves the highest Top-1 accuracy, while min-rank ensembles boost performance at higher Top-*k* thresholds. Notably, the ensemble of Llama 4 Maverick, DeepSeek Chat v3, and Gemini 2.0 Flash surpasses GPT-4.1 for relevant Top-5 and Top-10 settings with a 70% reduction in inference cost.

b Ensemble choices: min-rank 1 combines OpenAI GPT-4.1, Llama 4 Maverick, and Grok 3 mini Beta; min-rank 2 combines Llama 4 Maverick, DeepSeek Chat v3, and Gemini 2.0 Flash.

#### 2.1.2 LM Ensemble

We evaluate the suitability of an ensemble approach. Using performance on the validation set, we construct an ensemble of LMs comprising Grok 3 mini Beta, OpenAI GPT-4.1, and Llama 4 Maverick, and compare three aggregation strategies:

- Min-rank**:** assign each item the best rank it received across all models, promoting any item that at least one model ranks highly.
- Average-rank: compute the average rank across models, balancing contributions from all models and reducing the impact of any single outlier.
- Max-rank: assign each item the worst rank it received, ensuring only items consistently favored by every model appear at the top.

We observe that the min-rank and average-rank schemes substantially improve performance at Top-3, Top-5, and Top-10 (Figure 1), at the expense of a small drop in Top-1 accuracy compared to that of the best individual model. The high recall achieved by the ensemble arises from its diversity. Our observation is supported by the information-retrieval literature, where rank-fusion methods have been found to consistently improve recall by leveraging the complementary strengths of diverse rankers across topics and queries, demonstrating that greater diversity correlates with enhanced recall in ranking tasks.^47,48^ Consequently, min-rank and average-rank aggregation schemes are particularly effective for precursor prediction, unlike max-rank, they exploit the complementary strengths of diverse rankers and thus improve recall.

#### 2.1.3 Synthesis Condition Prediction

For the synthesis condition prediction, we evaluate LM performance in predicting parameters for a standard solid-state synthesis protocol involving two heating steps. First, precursor powders are mixed and homogenized to ensure a uniform distribution. Next, in the calcination stage, the precursor blend is calcined, activating thermal decomposition and diffusion that drive the formation of the target phase. Finally, during the sintering stage, elevated temperature promotes atomic grain–boundary and volume diffusion, which drives neck formation and growth, thereby consolidating and densifying the powder particles into a cohesive bulk body.^49,50^ In order to replicate experimental workflows, we prompt the LMs to predict calcination and sintering temperatures. The generated conditions are then compared against the curated 1000 entry subset of the data published by Kononova et al.^16^ We omit the associated dwell times here, as they are found to be strongly dependent on anthropogenic factors (e.g., an experimentalist’s preference to report 24 h over 19 h). Correspondingly, prior regression-based models tend to overfit to these factors rather than the underlying thermodynamics, resulting in poor predictive performance.^9,27^ In general, we note that LMs are fundamentally next-token predictors trained on a classification objective, supporting little inductive bias for regression.^51^ However, different works have focused on applying LMs to difficult numerical tasks with considerable success.^52^ In practice, synthesis temperatures are typically reported as integers (e.g., 800 °C), which are more accessible to the LMs.

Table 3 presents the results of our experiments, comparing the performance of LMs on the synthesis condition prediction task. For calcination temperature regression, OpenAI GPT-4.1 is the best-performing model, followed by Gemini 2.0 Flash and DeepSeek Chat v3. In the sintering temperature regression, Gemini 2.0 Flash achieves the best performance, with Llama 4 Maverick and OpenAI GPT-4.1 ranking next. Grok 3 mini Beta, previously second in precursor prediction, ranks among the lowest in both regression tasks.

**Table 3:** Synthesis Condition Prediction Performance^a^

	sintering temperature			calcination temperature		
model	MAE (↓)	RMSE (↓)	*R* ^2^ (↑)	MAE (↓)	RMSE (↓)	*R* ^2^ (↑)
ensemble avg. 1^b^	**96.31**	**134.48**	**0.667**	125.72	168.86	0.410
ensemble avg. 2^b^	96.89	135.42	0.663	**123.00**	**166.93**	**0.424**
						
Gemini 2.0 Flash	**100.66**	**142.22**	**0.628**	127.04	176.53	0.356
Llama 4 Maverick	102.76	145.23	0.612	135.85	180.90	0.323
OpenAI GPT-4.1	105.21	150.01	0.586	**125.92**	**174.45**	**0.371**
DeepSeek Chat v3	106.40	145.73	0.610	132.48	182.78	0.309
Mistral Small 3.1	113.93	156.36	0.550	137.05	185.20	0.291
Qwen 2.5 VL	131.93	174.06	0.443	142.68	192.72	0.232
Grok 3 mini Beta	131.00	175.56	0.433	152.09	205.97	0.123

a Regression performance for calcination and sintering temperature prediction.

b Ensemble composition: ensemble 1 comprises Gemini 2.0 Flash, Llama 4 Maverick, and DeepSeek Chat v3, ensemble 2 comprises OpenAI GPT-4.1, Gemini 2.0 Flash, and DeepSeek Chat v3. A parity plot can be found in Figure S4. Temperature values in °C.

MAEs vary considerably between the tasks, with 101 °C in predicting sintering temperatures compared with an MAE of 126 °C for calcination temperature regression. This is considerable given the fact that sintering temperatures are overall of higher magnitudes. We assess the source for the elevated error in calcination temperature predictions by benchmarking both the sintering and calcination tasks against a mean-predictor baseline (Table S3). We find that the normalized standard deviation of calcination temperatures is roughly 18% higher than that of sintering temperatures (Figure S2), suggesting that calcination is a more challenging target to learn. Moreover, calcination temperature may have a stronger dependence on factors not reported in the data set, such as variations in precursor particle size, a known factor in calcination conditions. For example, Pavlović et al. report that extending ball-milling duration for BaTiO_3_ by 1 h reduces the required calcination temperature by over 100 °C.^53^

Similar to the precursor recommendation task, we explore the performance of an ensemble of LMs by taking the average of the predictions of three LMs. For the sintering temperature regression, we use Llama 4 Maverick, Gemini 2.0 Flash, and DeepSeek Chat v3. Thereby we see a distinct performance improvement of 4% in *R* ^2^ over the best single-LM. For the calcination temperature regression, we add in a second ensemble by exchanging Llama with GPT-4.1. Notably, this setup is capable of increasing calcination temperature *R* ^2^ values by 5% to a reasonable 42.4%. Again, in close agreement with the precursor recommendation task, ensemble model configurations may reduce the inference cost by around 70% while boosting the performance.

We rationalize the underlying reason that an LM ensemble outperforms individual LMs. In materials synthesis, the mapping from processing recipes to target materials is inherently one-to-many.^29,54^ A single composition, such as BaTiO_3_, can be produced through multiple annealing protocols that vary in calcination and sintering conditions, most notably in temperature and dwell time. We generate a distribution of synthesis conditions and compare them to the literature-reported synthesis of 24 BaTiO_3_ samples. As shown in Figure 2, prompting individual LMs yields narrow distributions, with a single dominating mode peaked sharply around the means (orange, upper row). An LM ensemble substantially improves overlap with the ground-truth (purple). For example, in the case of calcination temperature, it correctly predicts a secondary mode below the mean while accurately reproducing the primary mode (blue, lower row). Similarly, for sintering temperature, the ensemble distribution captures the mean and additional features of the target distribution, such as a regime near 1200 °C. Most notably, for synthesis duration, individual LMs predict around the mean with a narrow spread, while the LM ensemble more accurately capture the long tail distribution at longer processing durations. As such, LM ensembles better capture one-to-many target-synthesis relationships, which offers a key insight into why they outperform individual LMs.

**Figure 2:**
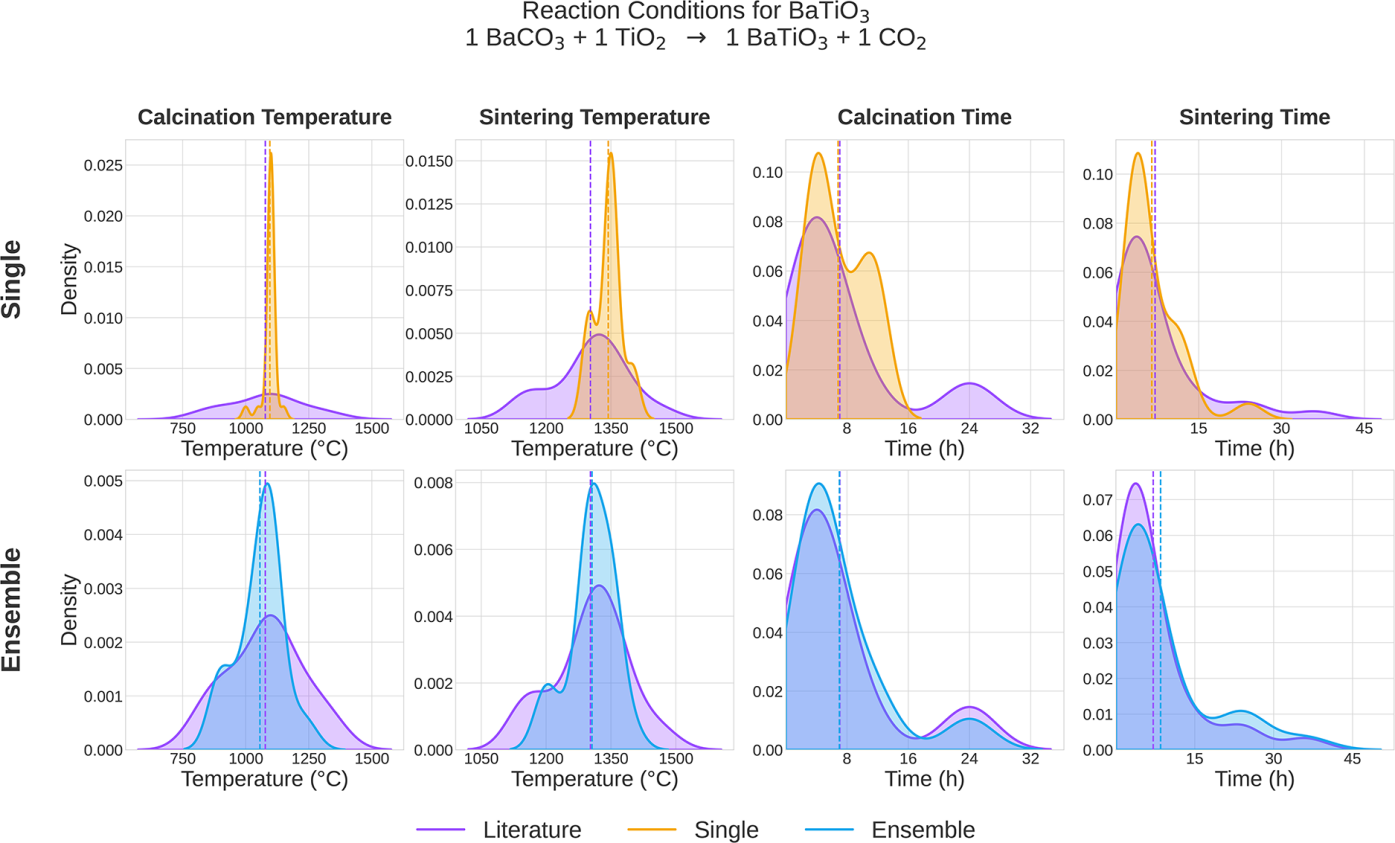
Synthesis condition distributions of literature-reported and LM generated solid-state synthesis recipes for BaTiO_3_. Literature distributions are shaded purple. Dotted lines refer to the mean value. “Single” refers to LM distributions acquired by drawing 24 samples from Gemini 2.0 Flash (orange). “Ensemble” refers to LM distributions acquired by sampling 8 predictions each from Llama 4 Maverick, DeepSeek Chat v3, and Gemini 2.0 Flash (blue). The individual LMs yield narrower distributions that fail to capture the underlying literature distribution, whereas the ensemble more accurately reproduces the literature’s secondary modes.

#### 2.1.4 Performance vs. Cost Trade-off

To compare the overall LM performances, we normalize each model’s performance to that of the best-performing model and compute the mean normalized score. We estimate costs using input and output token rates (Figure 3). GPT-4.1 and Gemini 2.0 Flash achieve the highest average performance. However, Gemini’s substantially lower price point makes it especially attractive for synthesis planning tasks. Notably, the ensemble of lower-priced models: Llama 4 Maverick, DeepSeek Chat v3, and Gemini 2.0 Flash outperform any single model while reducing cost by 70% relative to the top performing GPT-4.1. Moreover, as shown by our analysis, ensembles yield output distributions more closely aligned with the scientific literature, underscoring the joint cost and performance benefits.

**Figure 3:**
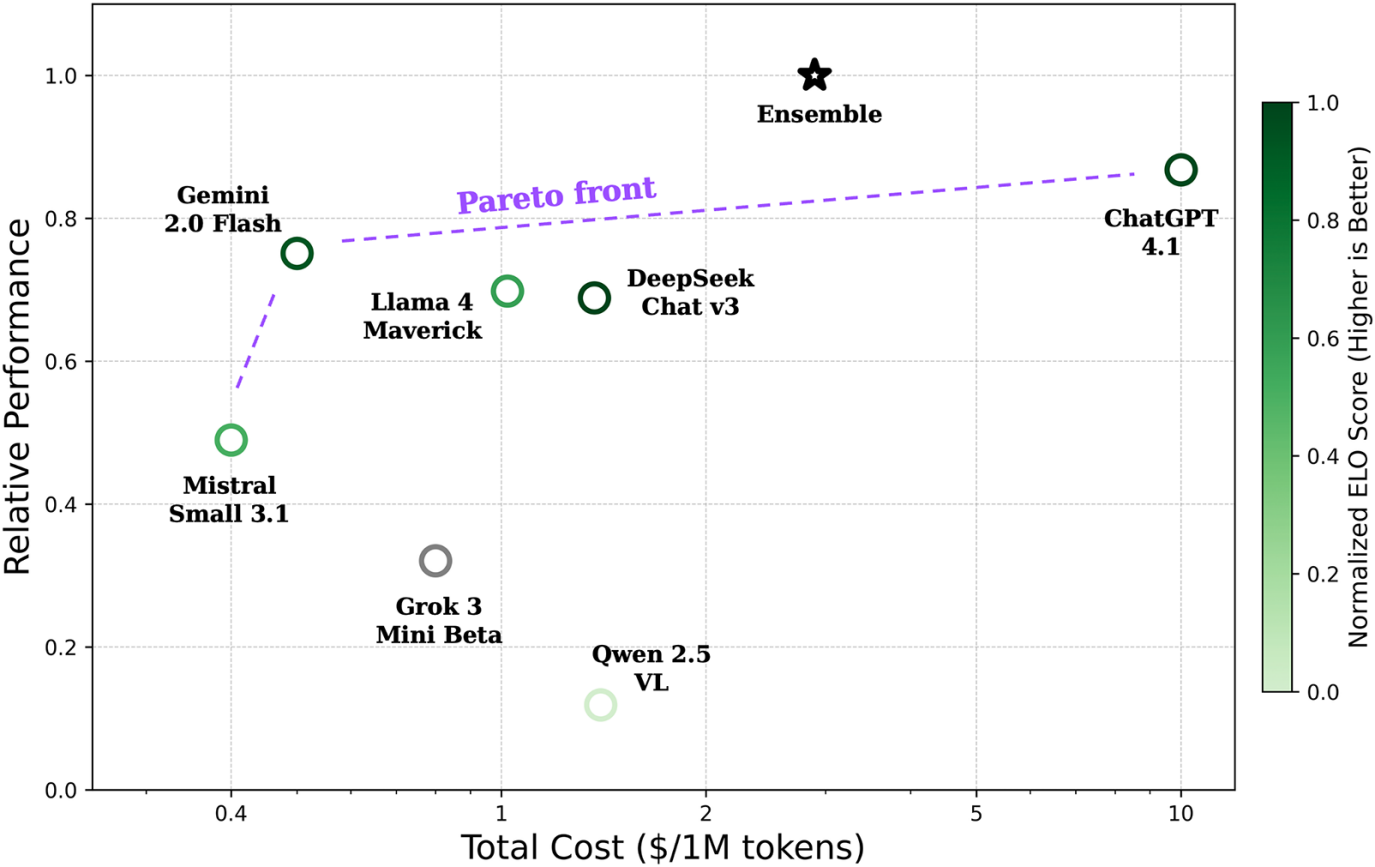
Comparison of model performance vs. cost. We compute each model’s relative performance on precursor prediction, calcination, and sintering temperature estimation tasks, and plot the average performance relative to cost. GPT-4.1 delivers the highest individual performance and comes at the highest cost. An ensemble of Llama 4 Maverick, DeepSeek Chat v3, and Gemini 2.0 Flash surpasses any single model in performance while reducing cost by 70% relative to GPT-4.1. The Elo rating score is represented by the color of each circle and serves as a quantitative indicator of model performance across common LM tasks.^55^ Cost estimates assume an equal proportion of input and output tokens, actual costs may vary because generated text length can differ across models.

**Figure 4:**
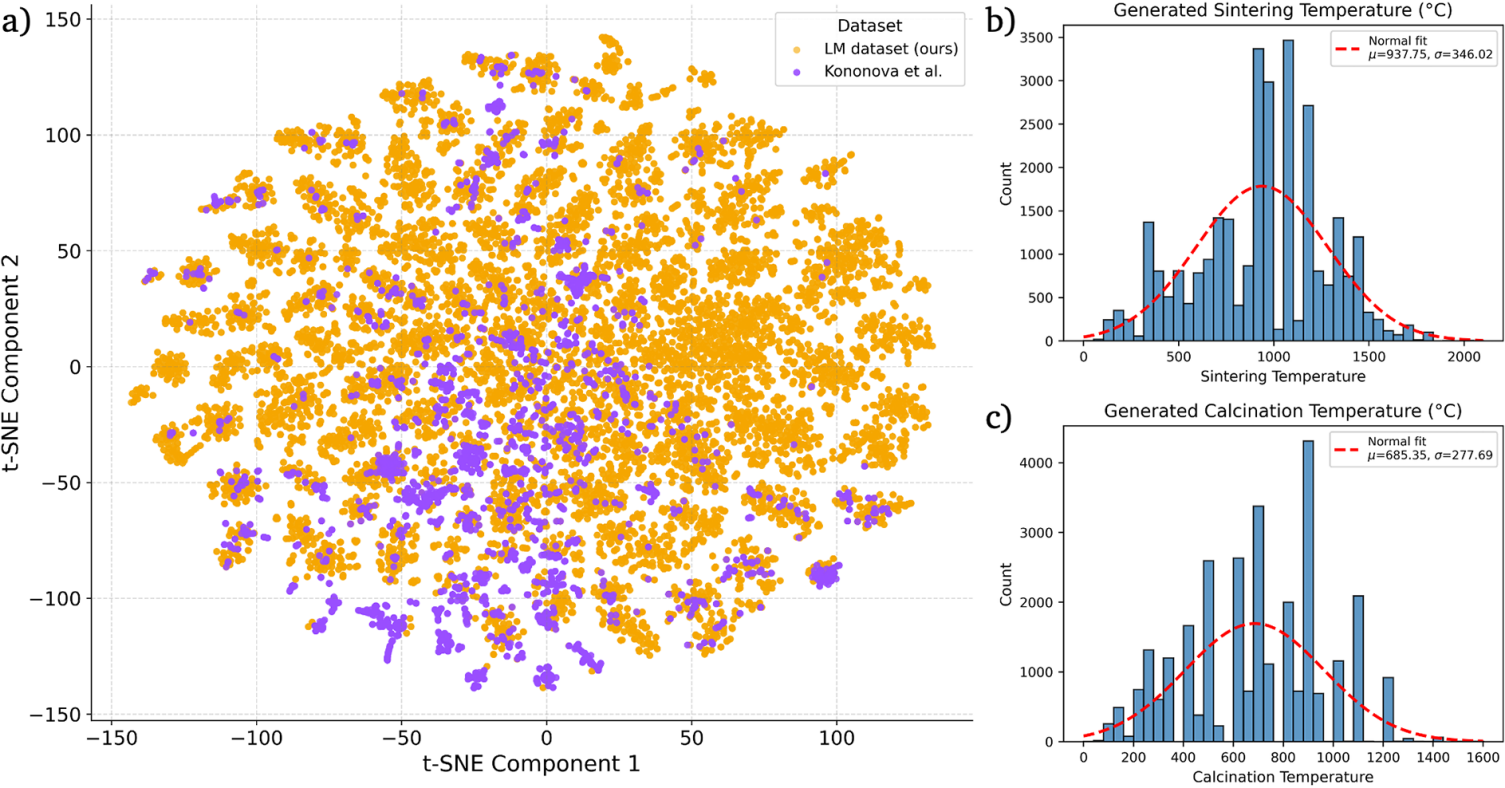
t-SNE projection of inorganic precursor compositions in embedding space. (a) Compositions from the Kononova data set^16^ are shown in purple, and LM-generated compositions in orange. Compositions are represented by standardized elemental-fraction vectors and projected via t-SNE. The expanded spread of orange points indicates that our generated data set spans a larger chemical composition space than the baseline data set. (b,c) Distributions of generated processing parameters.

### 2.2 Synthetic Data Augmentation Improves Model Performance

Our previous investigation demonstrated that LMs achieve strong performance in recalling already published synthesis parameters for solid-state synthesis. For LMs, true out-of-distribution evaluation on the 2 synthesis tasks cannot be assumed, as the training data have been publicly available for years or even decades and are therefore likely included in their training corpora. However, this result opens up the potential of leveraging LMs to generate synthetic data sets over synthesis parameter distributions to augment current size-limited experimental data sets. We evaluate the impact of LM-augmented data sets on state-of-the-art approaches in synthesis-condition prediction. This approach mirrors NLP strategies that augment scarce domain-specific data with LM-generated corpora (e.g., Xu et al.^56^) or employ teacher–student pseudolabeling frameworks.^57–59^ The rationale behind our methodology is to exploit the prior of LM learned estimates on synthesis conditions to warm-start our smaller expert models. We start by learning the LM estimates, which help our models learn the underlying broader trends, before we continue with training on experimental data.

Incorporating this workflow, we propose SyntMTE, a composition-based architecture derived from MTEncoder, a transformer model for representing inorganic materials, pretrained on the large Alexandria DFT database.^7,60^ Pretraining on large DFT corpora improves learned representations and downstream performance across materials-science tasks. As in NLP and computer vision,^61–63^ the pretraining objective need not be perfectly aligned with the final task, broad, physics-grounded supervision can still shape a model’s internal representation of chemical space in a way that transfers effectively. We therefore exploit the scale and coverage of publicly available DFT data sets, spanning millions of computed properties, to pretrain MTEncoder and then fine-tune it on the synthesis-related task, yielding consistent gains over models trained from scratch.

Our approach for modeling solid-state reactions extends on previous work^9^ by encoding both the reaction products and all associated precursors into embedding vectors. After embedding each material involved, we mean-pool a reaction representation and predict process parameters via multitask regression (Figure 5, right). We benchmark our approach on the Kononova data set^16^ using a time-based split, ranging from 2015 for training and using 2015–2016 for validation and later entries for testing. We introduce three baselines, a compositional feedforward network, a CrabNet-based transformer,^64^ and an XGBoost regressor^91^ with mean-pooled reaction features. The models are compared on three training regimes: a two-stage fine-tuning on synthetic and subsequently literature data, direct fine-tuning on literature data only, and training exclusively on synthetic data.

**Figure 5:**
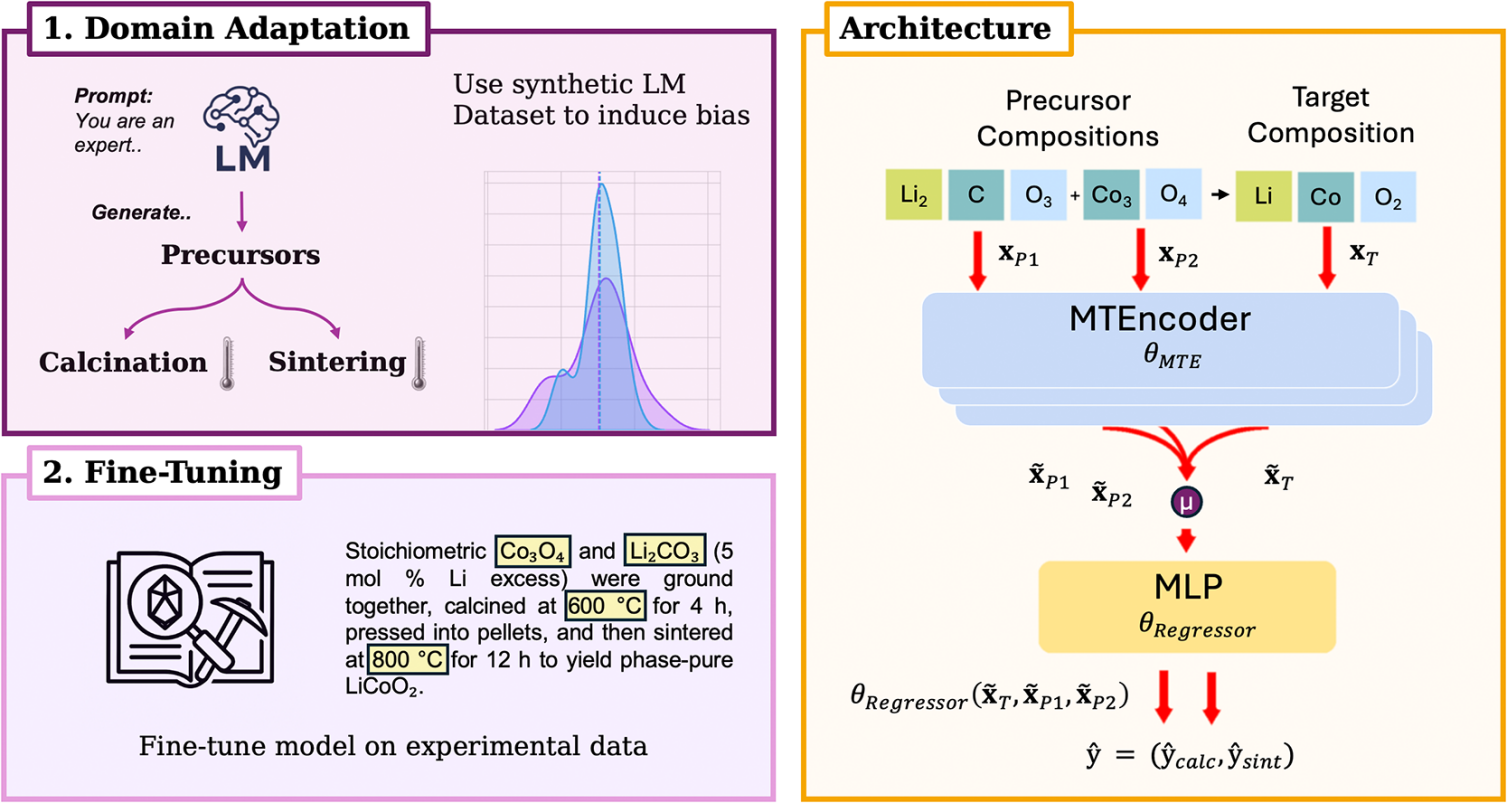
Overview of our synthesis-condition modeling. (Left) We first adapt the MTEncoder on a large LM-generated data set to bias it toward solid-state reaction conditions, then fine-tune on experimental literature recipes. (Right) Each precursor and the target composition are encoded by the shared MTEncoder (θ_MTE_) into embeddings ${\mathrm{x̃}}_{p,i},{\mathrm{x̃}}_{T}$ , pooled and concatenated, and passed through an MLP head (θ_Regressor_) to predict calcination and sintering temperatures $ŷ=\left({ŷ}_{calc},{ŷ}_{sint}\right)$ .

When trained exclusively on synthetic data, all models exhibit good performance despite having no exposure to literature-mined data and being trained only on recipes that do not overlap with the literature-based test set. Notably, the *R* ^2^ value, which measures the proportion of variance in the target variable explained by the model, remains high across all models except XGBoost. We hypothesize that XGBoost overfits the synthetic data, while also learning sintering and calcination separately, forfeiting the shared inductive bias of joint training, leading to lower performance, especially for calcination temperature prediction.

Finally, we evaluate the performance under both fine-tuning regimes. Across all models, except XGBoost, augmenting the training set with synthetic data consistently enhances performance, as evidenced by the relative MAE improvements in Table 4. This effect is most pronounced for SyntMTE, which achieves an MAE improvement exceeding 4% across both regression tasks. CrabNet attains a 3.8% improvement. The compositional feed-forward network likewise realizes improvements of 1%, surpassing the performance of the experimental data-trained SyntMTE model.

**Table 4:** Model Comparison^a^

			sintering temperature			calcination temperature			rel. MAE imp %
model	synth. data	literature data	MAE ↓	RMSE ↓	*R*2 ↑	MAE ↓	RMSE ↓	*R*2 ↑	↑
SyntMTE	√	√	135.00 (0.84)	181.30 (0.71)	0.545 (0.004)	153.72 (0.54)	199.71 (0.48)	0.436 (0.003)	4.08
SyntMTE		√	141.00 (2.13)	189.27 (2.46)	0.504 (0.013)	160.00 (2.97)	206.56 (2.53)	0.395 (0.015)	0.00
SyntMTE	√		149.43 (3.53)	197.78 (1.47)	0.428 (0.014)	169.63 (2.46)	214.64 (1.87)	0.358 (0.019)	–6.00
CrabNet	√	√	148.03 (1.00)	196.88 (1.60)	0.464 (0.009)	159.67 (0.89)	206.50 (1.13)	0.397 (0.007)	3.77
CrabNet		√	152.87 (6.59)	205.37 (8.20)	0.416 (0.047)	166.88 (3.13)	215.80 (0.46)	0.340 (0.025)	0.00
CrabNet	√		160.41 (2.12)	199.83 (0.69)	0.402 (0.016)	172.54 (4.07)	216.66 (1.47)	0.329 (0.036)	–4.13
Composition + NN	√	√	149.75 (0.87)	191.68 (0.78)	0.492 (0.004)	162.82 (0.41)	208.44 (0.50)	0.385 (0.003)	0.96
Composition + NN		√	150.23 (2.43)	193.82 (3.21)	0.480 (0.017)	165.38 (3.41)	211.54 (3.74)	0.366 (0.022)	0.00
Composition + NN	√		170.58 (2.74)	203.32 (0.06)	0.339 (0.016)	176.87 (3.56)	219.88 (0.00)	0.315 (0.017)	–10.09
Composition + XGBoost	√	√	163.62 (0.59)	210.47 (0.73)	0.387 (0.004)	179.54 (0.84)	228.00 (1.07)	0.263 (0.007)	–13.23
Composition + XGBoost		√	141.12 (0.90)	189.14 (1.45)	0.505 (0.008)	161.96 (0.87)	206.65 (1.05)	0.395 (0.006)	0.00
Composition + XGBoost	√		196.03 (1.90)	242.25 (1.83)	0.188 (0.012)	225.74 (3.49)	276.76 (3.48)	0.086 (0.027)	–39.16

a Comparison of embedding methods on different data regimes for sintering and calcination temperatures. We report the mean across five runs with standard deviation in parentheses. √ indicates training on the respective data source. Parity plots are presented in Figure S5. Temperature values in °C.

Our experiments show that the representation-learning models SyntMTE and CrabNet benefit most from data set augmentation. This can be seen in Figure S5, where we show the parity plots of two SyntMTE models, the literature only, and the augmented model. Overall, the comparison of models trained on literature-mined versus synthetic data reveals a significant opportunity to leverage synthetic data sets in synthesis modeling. Even training exclusively on large, LM-generated synthetic data sets can achieve good performance while eliminating the need for laborious manual data extraction. We also observe that the DFT-based pretraining objective of SyntMTE substantially improves performance when only limited literature data is available.

When the scores are compared to those of the best ensembles in the LM benchmark (see Table 3), the expert models exhibit lower performance across all regression tasks. Since LM benchmark scores may be inflated by data leakage, we argue that results cannot be directly compared to the year split-based predictions of SyntMTE, however, our results highlight the promising recent development of LM capabilities.

### 2.3 Application to Processing of LLZO Electrolyte Materials

Beyond assessing conventional performance-related material properties, virtual screening of compound-specific processing temperatures and durations offers a quantitative proxy for estimating manufacturing costs.^65^ As a case study, we consider the processing of solid-state electrolytes, which function as the replacement for conventional liquid electrolytes in next generation hybrid and future solid-state Li–ion batteries.^46,66^ Their key performance metrics are ionic conductivity and the electrochemical stability window.^44^ Although oxide-based electrolytes typically outperform alternatives in those metrics, they require densification through sintering at elevated temperatures when processed in the form of free-standing electrolytes (tape or pellet).^67^ One of the most promising material candidates among solid-state electrolytes is the garnet-type solid electrolyte Li_7_La_3_Zr_2_O_12_ (LLZO), which exhibits conductivities on the order of 1 × 10^–3^S cm^–1^ at room temperature. However, widespread integration into next-generation battery architectures is still hindered by high processing costs, which originate from precursor selection and the sintering protocols required to fabricate electrolyte tapes.^45^ Densification of cubic LLZO typically demands sintering at temperatures above 1050 °C for several hours, together with the incorporation of extrinsic phase-stabilizing dopants.^45,68–70^ Consequently, one of the most pressing challenges is to reduce the sintering temperature, a common requirement for high-value functional ceramics.^68^ Studies have demonstrated that aliovalent doping at the Li (A), La (B), and Zr (C) sites can lower the sintering temperature while stabilizing the desired cubic phase (Figure 6a).^68,71^

**Figure 6:**
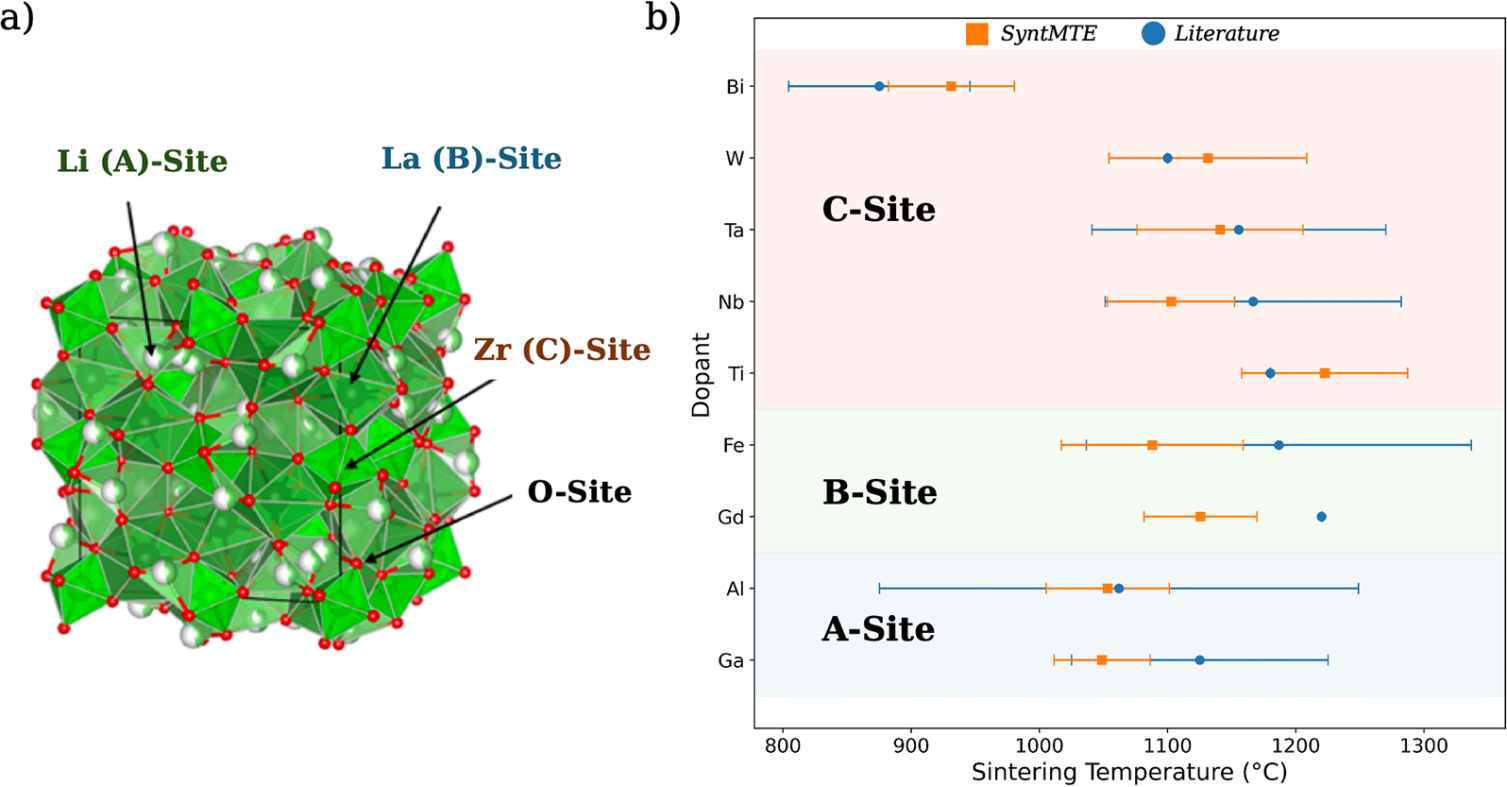
a) Probable doping sites in the cubic LLZO unit cell. Reproduced from reference 68. Available under a CC BY-NC-ND 4.0 license (https://creativecommons.org/licenses/by-nc-nd/4.0/). Copyright Mahbub et al.^68^ (b) True (blue circles) vs. predicted (orange squares) sintering temperatures with mean and standard deviation across different case reports per cation of the garnet electrolyte. Dopants grouped by crystallographic substitution site.

This interesting case provides an opportunity to evaluate the resolution of our method for predicting solid-state sintering temperatures. We curate a data set comprising 40 reported solid-state synthesis routes of doped LLZO variants in order to test if SyntMTE recovers compounds synthesizable at lower sintering temperatures. To test the extrapolation capabilities of our model, we withheld any LLZO-based compounds from the training sets and predicted the sintering temperatures of various doped LLZO compositions. Because literature-reported distributions are difficult to reconstruct precisely and several viable sintering temperatures exist per dopant family, we focus our analysis on the qualitative ordering of the compounds rather than on their exact values.

Among the C-site dopants investigated, tantalum is a well-studied yet comparatively moderate densification aid (Figure 6a). Supervalent substitution of Ta^5+^ for Zr^4+^ is charge-balanced by lithium vacancies (*V*_Li_), defect chemistry that accelerates lattice diffusion and stabilizes the cubic garnet framework.^72–74^ Experimentally, Ta-doped LLZO pellets are sintered for a few hours at 1100–1150 °C, about 100 °C lower than the temperature needed for undoped LLZO and intermediate between the behaviors of W- and Bi-doped compositions.^75^ Our model accurately reproduces the literature synthesis temperature window, predicting a mean temperature closely matching reported values and a standard deviation reaching approximately from 1060 to 1200 °C.

Substitution of Bi^3+^ with Zr^4+^-sites induces significant lattice expansion through the larger ionic radius of Bi^3+^ and yields higher lithium concentrations in the lattice, thereby enhancing ionic conductivity through larger migration channels, yielding a pronounced reduction in sintering temperature reported for a single-site dopant.^76,77^ Our model predicts a narrow sintering window for Bi of 880–980 °C, overestimating the minimum slightly yet accurately capturing the sharp decline to approximately 900 °C observed experimentally. For results at A- and B-site dopants, we observe Al spanning a broad sintering window owing to its small ionic radius and mixed-site occupancy, which promote gradual lattice relaxation and progressive vacancy clustering, thus smoothing the onset temperature.^78,79^ In contrast, Ga^3+^, whose ionic radius is moderately larger than native lattice cations, yields a narrower sintering profile. Our model’s prediction for Gd exhibits the largest deviation, being overly optimistic, whereas that for Fe aligns well with experimental values.

Overall, the results show that SyntMTE, despite receiving no prior training on LLZO, reproduces the key sintering temperature trends, underscoring the potential of synthesis planning models to guide future compound selection via the virtual screening of processing temperatures.

## 3 Discussion

Using LMs to propose synthesis parameters shows promise. However, our benchmarks center on solid-state synthesis, one of the most common methods in inorganic chemistry.^27^ This likely resulted in prior exposure of LMs to similar or the same synthesis routes we employ during benchmarking. Assessing the true extrapolative abilities remains challenging because genuinely novel routes are published infrequently, and the composition of LM training corpora is opaque. Ultimately, rather than using LMs as the final predictor, we argue that domain-specific models remain highly valuable. They can be fitted to a laboratory’s experimental outcomes, updated as new campaigns conclude, and deployed efficiently thanks to their smaller parameter counts. Our findings further indicate that LMs function most effectively as priors for smaller models. Via lightweight prompting, they can generate large data sets, which can be used to align domain-specific models while keeping computational costs low. Our generated data set should be interpreted in this light and applied with caution. Its validity is expected to be comparable to or below the benchmarked performance on precursor selection and synthesis-condition prediction. We constructed this data set solely to expose compact models to the LM-derived priors over synthesis parameters across the chemical space and did not intend it to represent true ground-truth protocols or strictly imply realizable synthesis routes. Furthermore, we did not evaluate LM fine-tuning in this study. Nevertheless, state-of-the-art LMs already generate useful precursor candidates and conditions when conditioned via several in-context examples, without task-specific parameter adaptation, offering a practical entry point. The outlined caveats, especially the potential data leakage, warrant caution when interpreting accuracy and motivate future work on targeted adaptation and systematic evaluation on domain coverage and uncertainty, as well as the comparison of large prompt-tuned versus smaller fine-tuned models.

## 4 Conclusions

Existing methods in ML-based material synthesis planning remain limited by the available training data. We demonstrate that current LMs can overcome this shortcoming. We benchmark seven state-of-the-art models on two standard tasks: precursor recommendation and synthesis condition prediction. Models such as GPT-4.1, Gemini-2.0, and Llama 4 Maverick achieve top-1 exact match accuracies for precursor prediction above 50%, rising to approximately 66% for their top five suggestions. We find ensembles of LMs enhance performance further by accurately capturing the synthesis windows, meaning the multitude of processing conditions enabling the synthesis of the same target compound. In contrast, individual LMs typically yield narrower, unimodal distributions, which reflect the synthesis window less accurately. Additionally, ensembles can reduce the inference costs by as much as 70%. We then employ LMs to distill materials-related knowledge into a synthetic solid-state synthesis data set containing nearly 28,548 complete recipes. To quantify the utility of this synthetic augmentation, we develop SyntMTE, a transformer fine-tuned in two steps, first on the synthetic LM data and second on literature-based extracted data. SyntMTE outperforms existing baselines, including state-of-the-art CrabNet. Compared with training on experimental data alone, our two-step training reduces the MAE for both sintering and calcination temperatures by approximately 6 °C. In a case study, we apply our framework to doped variants of LLZO, a solid-state electrolyte whose scalability is limited by an energy-intensive sintering process. Without LLZO-specific training, our model reproduces the broad sintering windows and captures the processing effects. This includes the substantial reduction in the sintering temperature achieved by Bi-substitution. These results demonstrate the model’s potential to identify low-temperature processing routes, for example, during the screening of novel compounds. Collectively, our study confirms that language-model-based methods can generate high-quality, cost-, and time-efficient auxiliary data on readily reported parameters and phenomena throughout the inorganic materials synthesis literature. This capability is critical as data remain scarce across the domain. Ultimately such models may be used to inform Bayesian optimization and guide autonomous experimentation, thereby accelerating the discovery and scalable production of advanced materials.

## 5 Methods

### 5.1 Data Set Preparation

We use the inorganic synthesis data set curated by Kononova et al.,^16^ which contains 33,343 text-mined solid-state synthesis recipes extracted from the scientific literature. After filtering for elemental consistency and removing ambiguous entries, 18,804 reactions remain, of which 9,255 are unique target–precursor pairs. For the precursor recommendation task, we randomly sample 1,000 entries for our LM evaluation. For synthesis condition prediction, we filter the data set for entries containing complete calcination and sintering temperature information, yielding another 1,000 entry subset. Both test sets are held out from the in-context examples. For the experiments on SyntMTE, we split the data chronologically, the training data include reactions reported up to 2014, the validation data spans 2015–2016, and the test data cover later reaction entries.

### 5.2 Language Model Evaluation

We evaluate seven state-of-the-art LMs via the OpenRouter API: OpenAI GPT-4.1, Google Gemini 2.0 Flash, Meta Llama 4 Maverick, xAI Grok 3 mini Beta, DeepSeek Chat v3, Alibaba Qwen 2.5 VL, and Mistral Small 3.1. Given logits *z*_*i*_ over the vocabulary V at step *t*, the next-token distribution is the temperature-scaled softmax

1 $$p_{\tau }\left(i|h_{t}\right)=\frac{\exp\left(z_{i}/\tau \right)}{\sum_{j\in V}\;\exp\left(z_{j}/\tau \right)}$$

where τ → 0 approaches greedy decoding and τ = 1 recovers the model’s native distribution. For our experiments, we use τ = 0.1 to produce near-deterministic outputs while retaining minimal stochasticity for parser retries. Each task uses 40 in-context examples sampled from the validation set. Prompts, which are available in the repository and Supporting Information, explicitly specify the required output format and include comprehensive chemical validity constraints. We employ a parser-aware retry policy, where up to three attempts per query are made, and queries still failing are marked as parsing failures and counted as false predictions.

#### 5.2.1 Precursor Recommendation Task

To ensure a consistent comparison, all generated precursor sets are normalized to canonical chemical formulas using pymatgen. Precursor sets are treated as order-invariant, and duplicates are removed across the 20 generated suggestions per target. Generated precursor sets are then compared against ground-truth precursors. We evaluate performance using a Top-*k* exact-match metric for *k* ∈ {1, 3, 5, 10}. Let *N* be the number of targets. For target *i*, let the ground-truth precursor set be *G*_*i*_ and the model’s ranked suggestions be (*S*_*i*,1_, ..., *S*_*i*,K_). All sets are canonicalized as described above. The Top-*k* exact match accuracy measures the fraction of targets for which the ground-truth precursor set appears among the top *k* predictions

2 $$EM@k=\frac{1}{N}\sum_{i=1}^{N}1\left[\exists j\leq ks.t.S_{i,j}=G_{i}\right],,k\in \left\{1,3,5,10\right\}$$

#### 5.2.2 Synthesis Condition Regression Task

For temperature prediction, we prompt models to output calcination and sintering temperatures in °C with 40 in-context examples. The outputs are parsed via regex. We report MAE, RMSE, and *R* ^2^. Outputs are returned as structured JSON, parse failures are retried up to three times, and residual failures are logged. Formally, let *y*_*i*_ denotes the ground-truth temperature and ${ŷ}_{i}$ denotes the model prediction for example *i*, let $\mathrm{y̅}=\frac{1}{N}\sum_{i=1}^{N}y_{i}$ , with *N* evaluated examples.

3 $$MAE=\frac{1}{N}\sum_{i=1}^{N}\left|{ŷ}_{i}-y_{i}\right|$$

4 $$RMSE=\sqrt{\frac{1}{N}\sum_{i=1}^{N}{\left({ŷ}_{i}-y_{i}\right)}^{2}}$$

5 $$R^{2}=1-\frac{\sum_{i=1}^{N}{\left({ŷ}_{i}-y_{i}\right)}^{2}}{\sum_{i=1}^{N}{\left(y_{i}-\mathrm{y̅}\right)}^{2}}$$

#### 5.2.3 Ensemble Methods

We construct ensembles using the models’ predictions across the same 1000 sample data sets introduced for the individual models. The ensemble approach leverages the diversity of different model architectures and training corpora to improve the overall performance and calibration. For the precursor recommendation, we aggregated ranked lists using three rank-fusion strategies. Let *r*_m_(*i*) denote the rank of candidate *i* assigned by model *m*. We evaluate three fusion methods: (i) min-rank *S*(*i*) = min_m_ *r*_m_(*i*), which promotes items that any model ranks highly; (ii) average-rank $S\left(i\right)=\frac{1}{M}\sum_{m}r_{m}\left(i\right)$ , which balances contributions from all models, and (iii) max-rank *S*(*i*) = max_m_ *r*_m_(*i*), which ensures only items consistently favored by every model appear at the top. For regression tasks, we aggregated temperature predictions by taking the mean across ensemble members.

### 5.3 Synthetic Data Generation

To assemble a diverse data set, we query the Materials Project,^6^ yielding 48,927 lab-synthesized compounds. We apply maximum-entropy sampling to select 10,000 target compositions, maximizing the estimated entropy of the selected set under featurization via MTEncoder representations.^60^ First, we prompt GPT-4.1 to flag and remove materials not synthesized via solid-state methods. Next, we generate precursor sets for each remaining target composition. In line with previous findings, we preserve the top three predictions per material, given the model’s robust Top–3 accuracy of 64.1% (Table 2). We then predict synthesis parameters, producing 29,473 entries, excluding generated routes found to be incomplete. Incorporating minimum temperature thresholds of 300 °C for calcination and 500 °C for sintering yields 28,548 plausible solid-state recipes. Figure 4a shows the enhanced compositional diversity of the generated data set when compared with the literature-mined data set by Kononova et al.^16^

### 5.4 SyntMTE Model Architecture and Training

SyntMTE is a transformer-based model derived from the MTEncoder framework,^60^ pretrained on the Alexandria DFT database^7^ across 12 materials properties (Table S5). It encodes each reaction by processing the target composition and all precursor materials with shared MTEncoder weights; the resulting embeddings are mean-pooled and concatenated, then passed to a two-layer MLP head for multitask regression of calcination and sintering temperatures. We fine-tuned all weights. Training uses Adam (learning rate 4.39 × 10^–5^) with L1 loss, batch size 25, and 200 epochs, the encoder hidden dimension is 512. Experiments were run five times each on two NVIDIA RTX A6000 GPUs.

### 5.5 LLZO Case Study Methodology

We study processing temperatures for LLZO garnet solid electrolytes. Reference LLZO literature is drawn from the corpus compiled by Mahbub et al.^68^ To ensure strict extrapolation, we exclude from training and validation any record whose target mentions LLZO or a commonly doped variant (Al, Ga, Ta, Nb, and W). Calcination and sintering temperatures were extracted using OpenAI’s o3 model, followed by manual spot checks. The SyntMTE model used for evaluation was fine-tuned sequentially on our synthetic recipes and then on the literature-mined corpus,^16^ after which it was applied to the mined LLZO data set. In Figure 6b, error bars aggregate across distinct literature routes and precursor choices, they reflect the across-route variability and do not represent model uncertainty.

## Supplementary Material



## Data Availability

The source code underlying this project is available at the GitHub repository https://github.com/Thorben010/llm_synthesis.

## Notes

Extended methods and data set curation; prompting templates and in-context examples; evaluation protocols; additional benchmarking tables and ablations; and prompt listings and reproducibility notes (PDF)
