# Supplementary material for: Language Models Enable Data-Augmented Synthesis Planning for Inorganic Materials
Source: ACS Appl Mater Interfaces. 2025 Nov 26;17(51):69221–33. doi: 10.1021/acsami.5c11229 (PMC12754754; doi:10.1021/acsami.5c11229)
Supplement: Supplementary file 1 [file am5c11229_si_001.pdf]

Supporting Information: Language Models Enable Data-Augmented Synthesis Planning for Inorganic Materials

Thorben Prein[†,‡,¶], Elton Pan[§], Janik Jehkul[‖], Steffen Weinmann[†], Elsa Olivetti[§], and Jennifer L.M. Rupp[*,†,¶]

[†]School of Natural Sciences, Technische Universität München, Garching b. München, 85748, Germany.

[‡]Munich Data Science Institute, Garching b. München, 85748, Germany.

[¶]TUMint. Energy Research GmbH, Garching b. München, 85748, Germany.

[§]Department of Materials Science and Engineering, Massachusetts Institute of Technology, Cambridge, Massachusetts 02139, United States.

[‖]School of Computation, Information and Technology, Technische Universität München, Garching b. München, 85748, Germany.

*Corresponding author: jrupp@tum.de

S1 Prompting Strategy

In-context learning refers to a language model's ability to solve a novel task from prompt-side demonstrations without parameter updates. Performance in this setting typically improves with the number of examples provided. This phenomenon was first highlighted in GPT-3, where accuracy on benchmarks increased as the number of demonstrations was varied from 1–32, demonstrating that richer prompts can substitute for task-specific fine-tuning.[80] Here, we adopt this methodology to determine an appropriate number of examples for our models. Our evaluation using Mistral Small on the precursor suggestion task shows that overall performance improves up to 40 in-context examples and plateaus beyond this point. Accordingly, we use 40 held-out examples in our evaluations on all models.

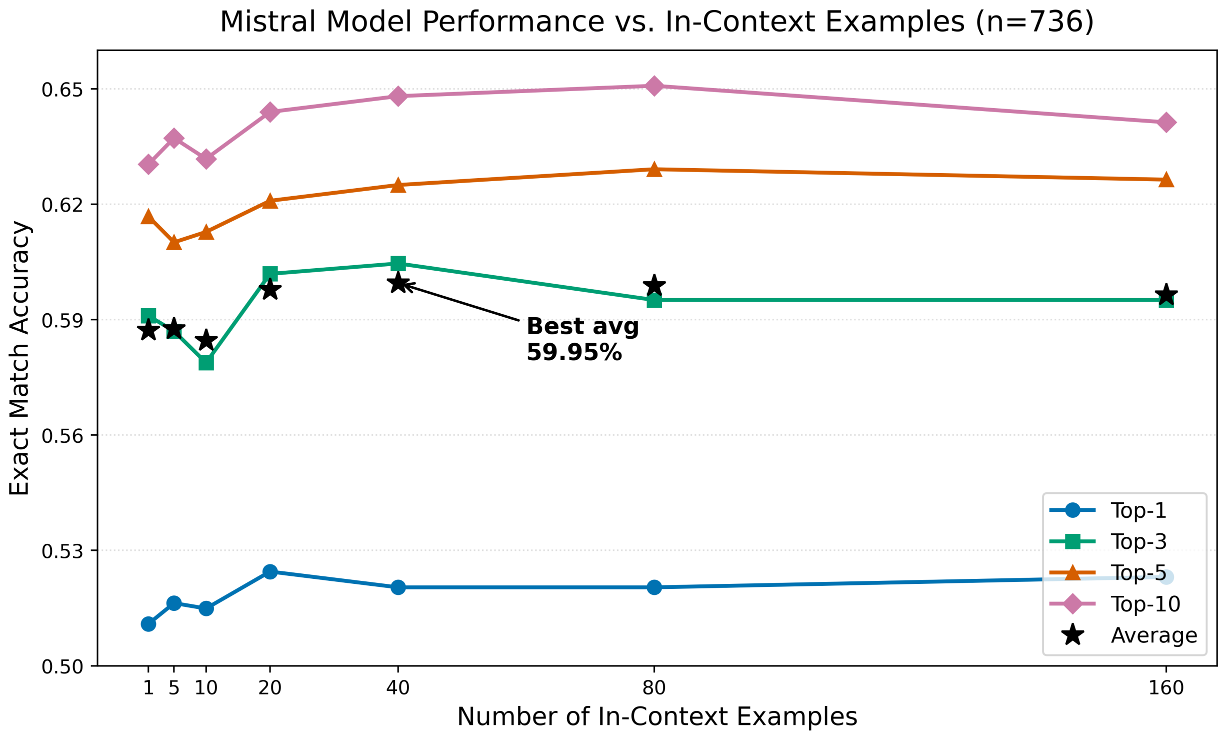

Figure S1: Mistral Small 3.1 performance as a function of the number of in-context examples, evaluated on 736 samples from the validation set. The best average performance is achieved with 40 in-context examples.

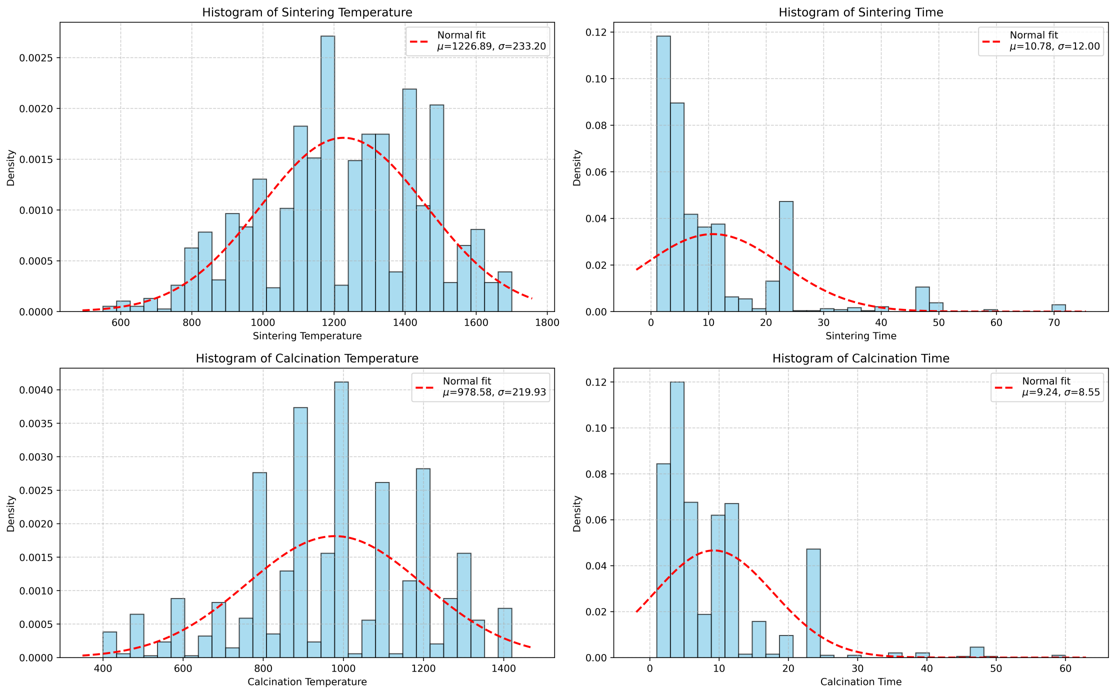

Figure S2: Statistics for the synthesis-condition test set used in our evaluation.

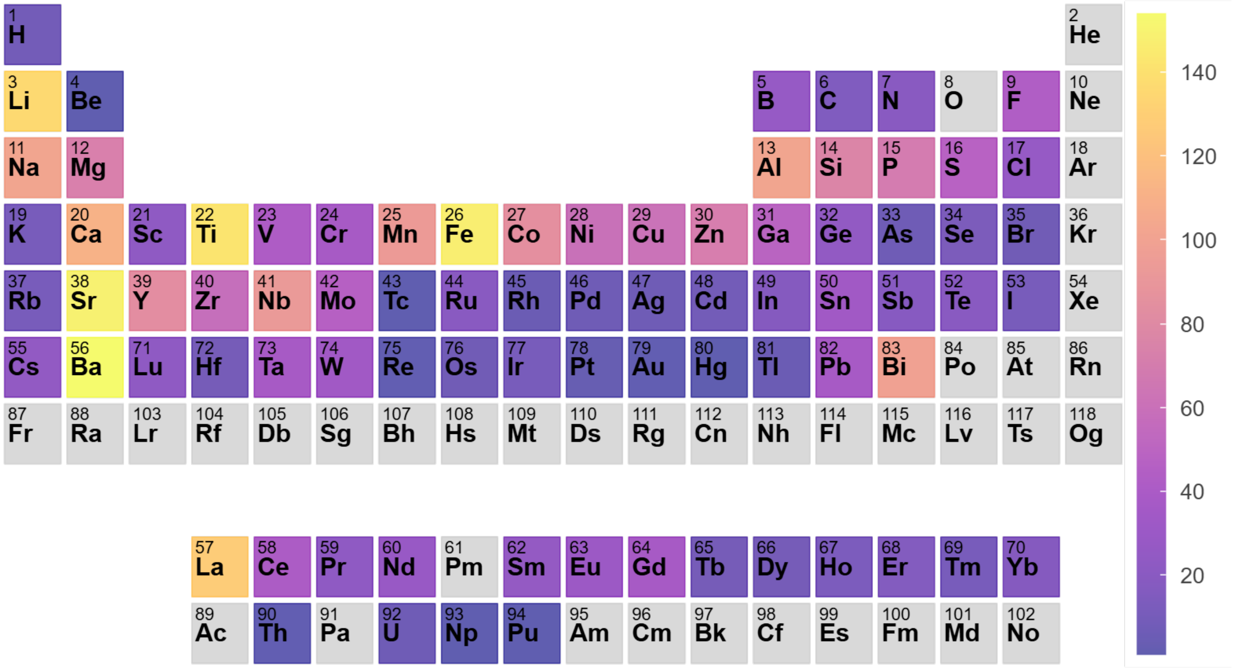

Figure S3: Periodic table heatmap illustrating the frequency distribution of elements within the target material formulas (oxygen excluded).

S2 Benchmarked Language Models

In our study, we systematically evaluated seven contemporary LMs, selected to represent a diverse array of architectures, parameter scales and licensing schemes. The main characteristics of these models are summarized in Table S1.

Table S1: LMs evaluated in this work, sorted by release date. Arena scores from the Chatbot Arena leaderboard as of May 18, 2025

Model	Release	#Params	#Active	Context Window	Open Source	ELO Score	MMLU-Pro
Qwen 2.5 VL-72B	Jan 2025	72B	-	32k tokens	Yes (Qwen License)	1123	71.2
Mistral Small 3.1	Mar 2025	24B	-	128k tokens	Yes (Apache 2.0)	1249	66.8
DeepSeek-V3-0324	Mar 2025	671B	37B	128k tokens	Yes (MIT License)	1369	81.2
Gemini 2.0 Flash	Feb 2025	-	-	1M tokens	No	1352	76.4
GPT-4.1	Apr 2025	-	-	1M tokens	No	1365	-
LLaMA 4 Maverick	Apr 2025	400B	17B	1M tokens	Yes (LLaMA 4 Comm. License)	1266	80.5
Grok 3 Mini Beta	Apr 2025	-	-	131k tokens	No	-	78.9

Qwen 2.5 VL-72B. Released in January 2025, Qwen 2.5 VL-72B (Apache 2.0 license) comprises 72 billion parameters and features a context window of 32,000 tokens. The model achieved an ELO rating of 1123 and an MMLU-Pro score of 71.2.[81,82]

Mistral Small 3.1. This model was introduced in March 2025 under the Apache 2.0 license and contains 24 billion parameters. It offers an extended context window of 128,000 tokens. While detailed training data information remains undisclosed, it attains an ELO score of 1249 and an MMLU-Pro score of 66.8, indicating robust generalization abilities.[82,83]

DeepSeek-V3-0324. This model, released under the MIT license, is a Mixture-of-Experts (MoE) language model featuring 671 billion total parameters, with 37 billion activated per token. Its MoE layers consist of 1 shared and 256 routed experts, with 8 routed experts actively engaged for each token. It's pre-trained on 14.8 trillion tokens and supports a context window extended to 128,000 tokens. The March 2025 checkpoint of this model achieves the highest ELO (1369) and MMLU-Pro (81.2) score among the evaluated models.[82,84,85]

Gemini 2.0 Flash. Released in February 2025 as a proprietary model, Gemini 2.0 Flash features a context window of 1 million tokens. While the specific parameter count and training details are not publicly disclosed, the model's performance is notable, reflected by an ELO rating of 1352 and an MMLU-Pro score of 76.4.[82,86]

GPT-4.1. This model was introduced in April 2025, also supporting a large context window of 1 million tokens. Despite undisclosed training and parameter specifics, it achieved a remarkably high ELO score of 1365.[82,87]

LLaMA 4 Maverick. The model, released in April 2025 under the LLaMA 4 Community License, incorporates a Mixture-of-Experts (MoE) architecture with 128 experts. It features a grand total of 400 billion parameters, of which approximately 17 billion are actively engaged during inference. Trained on an extensive dataset of roughly 22 trillion tokens, its 1 million

token context window supports advanced in-context learning capabilities. Evaluation metrics include an ELO score of 1266 and an MMLU-Pro score of 80.5.[82,88]

Grok 3 Mini Beta. This proprietary LM, launched in April 2025, provides a context window of 131,000 tokens. It attained an MMLU-Pro score of 78.9. The ELO score was not disclosed publicly.[89]

S3 Multi-Provider Inference via OpenRouter Proxy

All model inference is performed through the OpenRouter API, which federates requests to multiple upstream providers under a single authentication and billing framework. For every model we set the temperature parameter at $\tau = 0.1$ and retain all other provider defaults. All models in Table S1 were benchmarked via OpenRouter on 1,000 held-out targets. These models were selected to represent a diverse cross-section of leading commercially available large language models, with a primary focus on balancing strong performance and cost-effectiveness.

Table S2: Per-token API costs via OpenRouter (May 23, 2025)

**Model**	**Input ($/1M)**	**Output ($/1M)**
GPT-4.1 (OpenAI)	2.0	8.0
Grok 3 Mini Beta (xAI)	0.3	0.5
Llama 4 Maverick (Meta)	0.17	0.85
DeepSeek Chat v3 (DeepSeek)	0.27	1.1
Mistral Small 3.1 (Mistral)	0.10	0.30
Gemini 2.0 Flash-001 (Google)	0.10	0.40
Qwen 2.5 VL 72B (Alibaba)	0.70	0.70

In our framework all API requests and responses are recorded in a structured log for full traceability. If a response doesn't satisfy the prescribed output format (missing the required list or dictionary structure), the identical prompt is retried up to two additional times, for a total of three format validation attempts. Should all three attempts yield structurally invalid output, the response is marked as a failure. API-level errors are immediately assigned a value of `None`. They are logged, and retried without counting against the three allowed format

attempts. This separation ensures that transient infrastructure issues do not penalize model performance estimates.

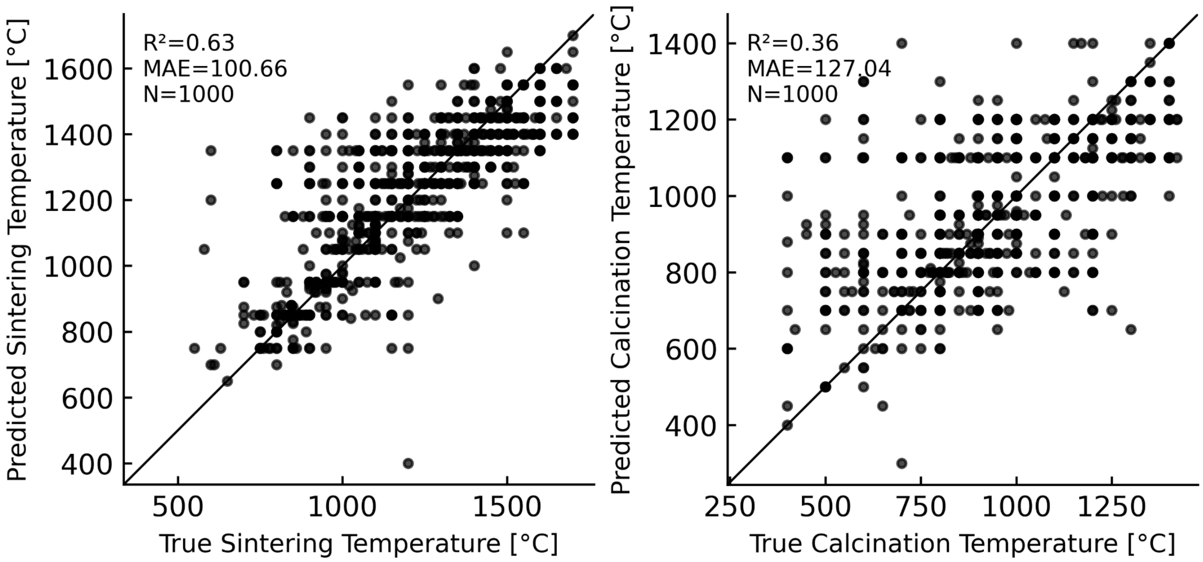

Figure S4: Scatter plot of true versus predicted sintering temperatures for the Gemini 2.0 Flash model on the 40-shot test.

Table S3: Mean predictor baseline: descriptive and error metrics for synthesis-condition parameters.

**Parameter**	**Mean**	**Std. Dev.**	**Norm. Std. (Std/Mean)**	**MAE**	**RMSE**
Sintering temperature	1,230	233	0.190	192	233
Sintering time	10.8	12.0	1.11	8.49	12.0
Calcination temperature	979	220	0.225	177	220
Calcination time	9.24	8.56	0.926	6.18	8.55

S4 Expert Baselines for Precursor Suggestion

We compare two representative approaches, for the precursor prediction task, which do not require large LMs and run locally. ElemwiseRetro adopts a template-based strategy: for each constituent element of the target, the model predicts a precursor template (e.g., carbonate, hydroxide) and then composes these per-element choices into a complete precursor set.[24] SynthesisSimilarity is a retrieval-style baseline that scores candidates by their

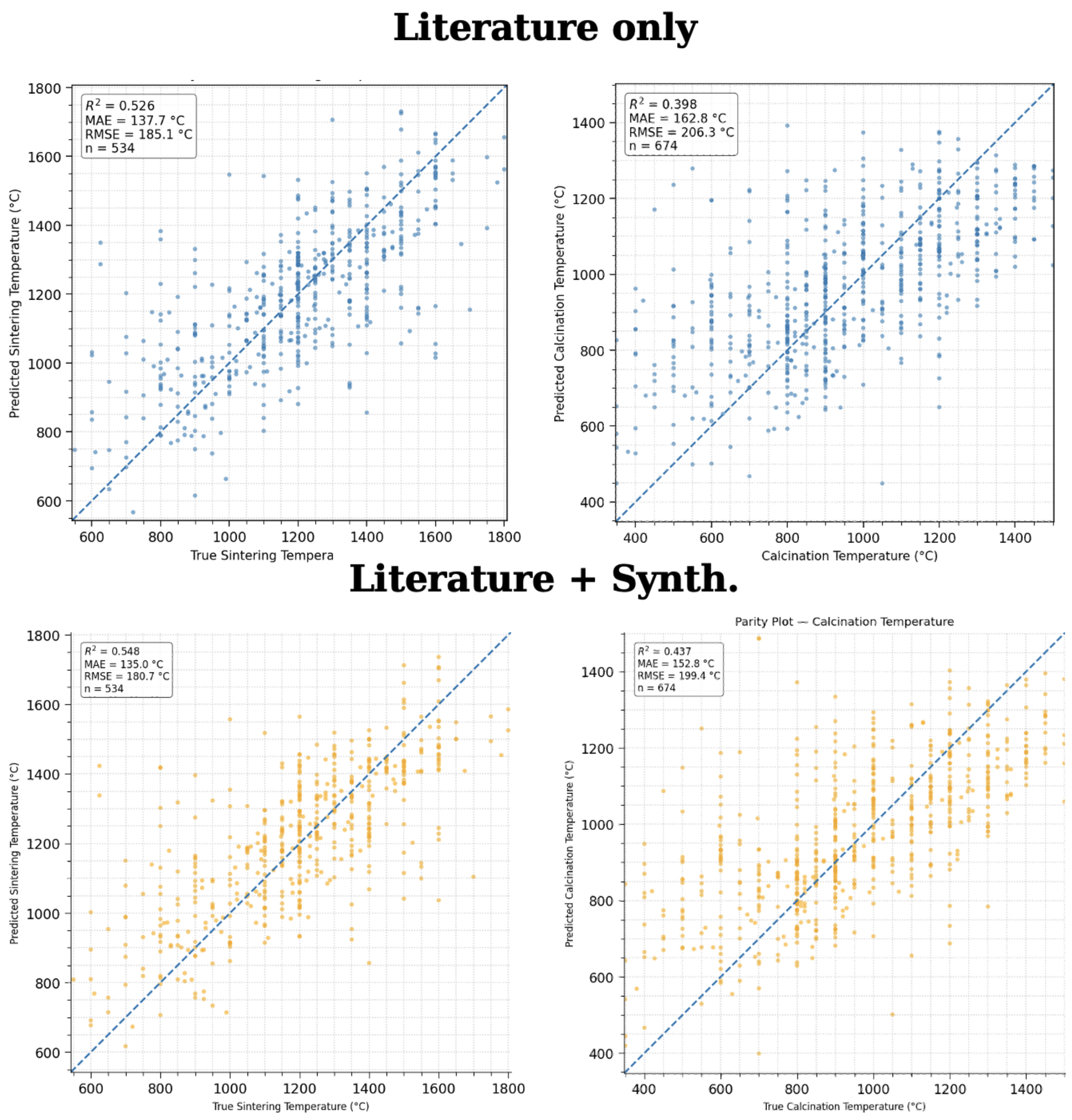

Figure S5: Parity plots for the regression task shown in Table 4. The literature only model has been trained only on literature data, while the model Literature + Synth. is sequentially trained on both datasets. Notably R2 scores are increased in the second setting.

proximity to known syntheses in a joint precursor and condition feature space, leveraging similarity to the reaction archive rather than learning a purely parametric mapping.[24]

We also compare the LM-based precursor suggestions with expert-based models. Notably, LMs perform strongly in this head-to-head comparison, likely for two reasons: (1) they may have memorized many solid-state reactions from their training data, and (2) they learn richer notions of materials similarity, enabling them to identify effective precursor pairings without

being constrained to the limited vocabularies of the baseline models described above.

For the baseline comparison, we partition the Kononova dataset into 70% training, 10% validation, and 20% test. To mimic a prospective setting, we apply a chronological split in which the test set contains only synthesis routes reported after 2014, while the training and validation sets are drawn from earlier records. Duplicate entries are removed following the procedure described in Noh et al.[25].

Table S4: Baseline performances on the Kononova dataset.[16]

**Model**	**Top-1**	**Top-3**	**Top-5**	**Top-10**
ElemwiseRetro[23]	48.72	55.19	57.30	59.08
SynthesisSimilarity[24]	40.40	53.48	57.61	61.24

S5 SyntMTE Experiments

For the regression task we employ several baseline models.

S5.1 CrabNet

CrabNet is a composition-only materials prediction framework that leverages a transformer encoder to learn contextualized embeddings for each element in a compound.[64] By combining learned element vectors with sinusoidal "fractional embeddings" of stoichiometry, CrabNet's multi-head self-attention layers capture complex inter-element interactions without any hand-crafted descriptors or structural information. This design not only yields state-of-the-art accuracy on benchmarks like MatBench, often outperforming graph-based models such as Roost,[90] but also enables interpretability: attention maps highlight which element pairs drive a given property prediction. In our experiments we use three transformer blocks.

S5.2 MTEncoder (`SyntMTE`)

Figure S6 depicts the MTEncoder workflow, demonstrating how a material's elemental representation is encoded via a transformer-based model.[60] Each material is broken down into individual element tokens (e.g., Na, Fe, O) alongside a dedicated "Compound" token (*CPD*) that aggregates the element-specific information. These tokens are fed into the transformer encoder, which produces context-rich embeddings for the composition. The embedding associated with the CPD token serves as the learned representation of the material and is passed to an MLP head to predict various properties. Pretraining is conducted using the Alexandria database on 12 tasks (Table S5[7]).

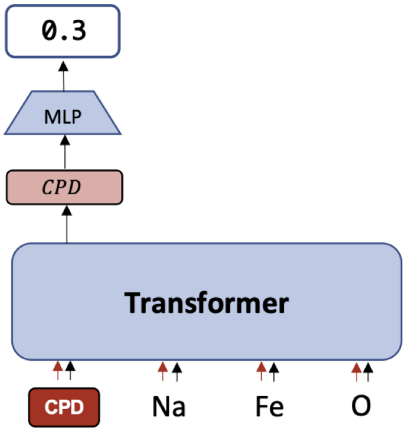

Figure S6: Overview of the MTEncoder model. Material compositions are tokenized and processed by a transformer to generate feature embeddings for downstream property prediction.

S5.3 Composition + NN

Three-layer feed-forward neural network: This model comprises three fully connected layers, each followed by a ReLU activation, optional dropout for regularization, and layer normalization to accelerate convergence and stabilize training. Compositional feature vectors in the form of elemental fractional embeddings are input to this multilayer perceptron to predict the target property.

Table S5: Pretraining objectives for MTEncoder. These tasks are drawn from the Alexandria materials dataset.[7]

**Pretraining Objectives**
Stress
Band Gap (Direct)
Band Gap (Indirect)
Density of States at Fermi Level
Energy Above Hull
Formation Energy
Corrected Total Energy
Phase Separation Energy
Number of Atomic Sites
Total Magnetic Moment
Crystal Space Group
Masked Element Reconstruction (Self-Supervised)

S5.4 Composition + XGBoost

We use gradient-boosted decision trees on composition-only features. Each formula is featurized with standard stoichiometric features, yielding a fixed-length vector per composition. XGBoost models nonlinear interactions among these descriptors via an ensemble of shallow trees and serves as a strong, fast baseline for synthesis-condition prediction.[91]

S5.5 Bias Analysis of SyntMTE

We investigate the literature-trained SyntMTE models' accuracy across chemistry clusters. Test MAEs span 91–197 °C, with the best performance in LLZO-like Li–La–Zr oxide clusters and the weakest in heterogeneous phosphate, borate, and silicate clusters. This spread tracks data density and chemical coherence. The model generalizes better within those families of closely related compositions, whereas sparsely sampled or chemically diverse clusters exhibit larger errors. Non-oxide and heavily doped compositions also show poorer transfer, consistent with limited representation and broader processing-temperature ranges.

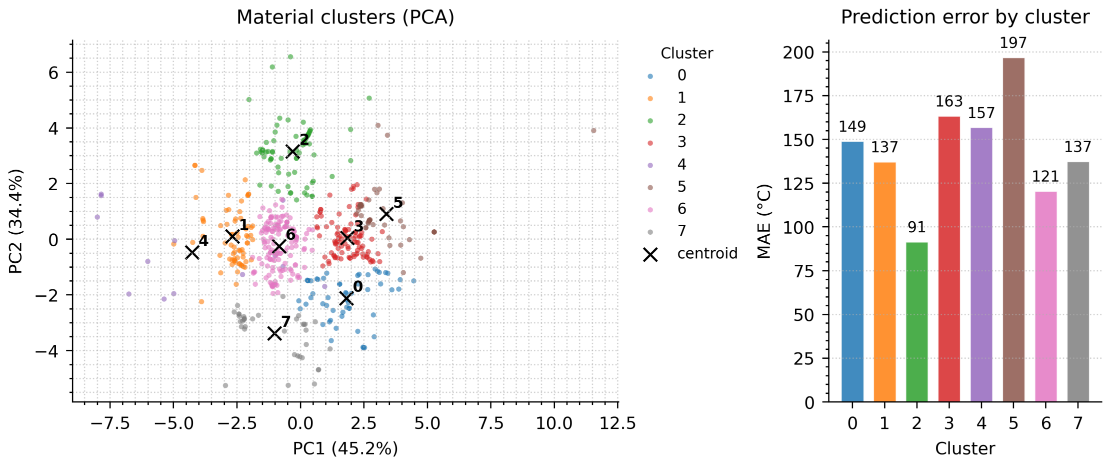

Figure S7: Unsupervised cluster analysis of the test set (k=8). Left: PCA embedding colored by cluster; crosses mark centroids. Right: per–cluster MAE for sintering–temperature prediction.

S6 Prompt Template

Listing 1 shows the full prompt used for predicting precursor combinations for the solid-state synthesis of $Na_3Bi(AsO_4)_2$. The prompt explicitly instructs the model to generate 20 plausible precursor combinations suitable for solid-state synthesis, ensuring that each combination contains all required elements from the target material, favors stable and common laboratory reagents and ranks combinations by chemical plausibility. We also leverage in-context learning, providing 40 examples embedded in the prompt (Figure S1).

```
You are a computational chemistry expert specializing in solid-state
    synthesis and retrosynthesis.
Your task is to identify potential precursor combinations for solid-
   state synthesizing the target material: 'Na3Bi(AsO4)2'.


**Requirements**:
1.  Generate 20 distinct combinations of precursor materials.
2.  Use standard chemical formulas ONLY (e.g., 'TiO2', 'Na2CO3').
3.  **Constraint Check:** Ensure each precursor combination contains
    ALL elements present in the target material 'Na3Bi(AsO4)2'.
```

```
   Assume Oxygen and other common laboratory elements (e.g., C for
   carbonate sources) are available.
4.  **Plausibility Filter:** Prefer chemically plausible routes
   using reasonably common and stable laboratory reagents. A
   plausible route is one that uses precursors commonly found in
   solid-state synthesis and avoids highly unstable or rare
   compounds.
5.  Order the 20 combinations from the MOST plausible/common
   synthesis routes to the LEAST plausible/common.
6.  **Common Precursor Types:** Consider oxides (e.g., TiO2, Fe2O3),
    carbonates (e.g., Na2CO3, CaCO3), nitrates (e.g., KNO3, Ca(NO3)
   2), hydroxides (e.g., Al(OH)3), and other standard laboratory
   reagents.
7.  **No Gases:** Do not include 'O2' in the precursor combinations.
8.  If the target material is not suitable for solid-state synthesis
   , respond with False as a boolean.

**Examples of Target -> Precursors:**
- Target: ' M o F ', Precursors: [False] #only synthesizable via gas-
   solid reaction, not suitable for conventional solid-state
   synthesis
- Target: 'Gd2TiO5', Precursors: ['TiO2', 'Gd2O3']
- Target: 'NdTl(MoO4)2', Precursors: ['MoO3', 'Tl2O3', 'Nd2O3']
- Target: 'Sr(GaO2)2', Precursors: ['SrCO3', 'Ga2O3']
- Target: 'La0.075Ta2O5.113', Precursors: ['La2O3', 'Ta2O5']
- Target: 'LaFeO3', Precursors: ['Fe2O3', 'LaCO3']
- Target: 'Sr1.9Ca1Tl0.9V0.1Cu2Bi0.1O7', Precursors: ['SrCO3', '
   Tl2O3', 'CuO', 'CaO', 'Bi2O3', 'V2O5']
- Target: 'La3RuO7', Precursors: ['RuO2', 'La2O3']
```

```
- Target: 'Zr0.8Ti1Sn0.2O4', Precursors: ['SnO2', 'TiO2', 'ZrO2']
- Target: 'La1Fe0.95W0.05O3', Precursors: ['WO3', 'Fe2O3', 'La2O3']
- Target: 'Bi3PO7', Precursors: ['PH9(NO2)2', 'Bi2O3']
- Target: 'CsTaWO6', Precursors: ['WO3', 'Cs2CO3', 'Ta2O5']
- Target: 'TaWO6', Precursors: ['WO3', 'Ta2O5']
- Target: 'Dy0.05Zn1Ga1.95O4', Precursors: ['Dy2O3', 'Ga2O3', 'ZnO']
- Target: 'Nd0.02Gd0.98V1O4', Precursors: ['Gd2O3', 'V2O5', 'Nd2O3']
- Target: 'Ba1Pr0.8In0.2O3', Precursors: ['BaCO3', 'Pr6O11', 'In2O3'
  ]
- Target: 'Ba4SrSmTi3V7O30', Precursors: ['SrCO3', 'Sm2O3', 'BaCO3',
   'TiO2', 'V2O5']
- Target: 'ZrSiO', Precursors: ['SiO2', 'ZrO2']
- Target: 'Ba0.6Sr0.4Nb0.1Co0.9O3', Precursors: ['SrCO3', 'Nb2O5', '
  BaCO3', 'Co2O3']
- Target: 'CsAlP2O7', Precursors: ['Cs2O', 'P2O5', 'Al2O3']
- Target: 'Ag4.64Pb2O5.87', Precursors: ['Ag2O', 'PbO']
- Target: 'Ca1Ti4Cu3.2O12', Precursors: ['CuO', 'TiO2', 'CaCO3']
- Target: 'Gd0.3Fe1Bi0.7O3', Precursors: ['Gd2O3', 'Bi2O3', 'Fe2O3']
- Target: 'YTiO', Precursors: ['TiO2', 'Y2O3']
- Target: 'Ba6Sn6Se13', Precursors: ['BaSe', 'Se', 'Sn']
- Target: 'V0.9Cu0.1Bi2O5.35', Precursors: ['CuO', 'Bi2O3', 'V2O5']
- Target: 'MgNb2(PbO3)3', Precursors: ['Nb2O5', 'MgCO3', 'PbO']
- Target: 'Cs0.75Rb0.25P1H2O4', Precursors: ['RbP(HO2)2', 'CsP(HO2)2
  ']
- Target: 'TiCdO3', Precursors: ['TiO2', 'CdO']
- Target: 'Pu0.9Am0.1O2', Precursors: ['AmO2', 'PuO2']
- Target: 'La0.9Mn1Pb0.1O3', Precursors: ['PbO', 'MnO2', 'La2O3']
- Target: 'Li3.55Ca5.45Si3O12.45F1.55', Precursors: ['SiO2', 'CaCO3'
  , 'Li2CO3', 'LiF']
```

```
- Target: 'HoMnO3', Precursors: ['Mn2O3', 'Ho2O3']
- Target: 'SiPbC', Precursors: ['Pb', 'SiC']
- Target: 'CsAl(SiO3)2', Precursors: ['SiO2', 'Cs2CO3', 'Al2O3']
- Target: 'CdIn2O4', Precursors: ['CdO', 'In2O3']
- Target: 'CdWO4', Precursors: ['WO3', 'CdO']
- Target: 'Sr1.8Ca0.9Ti0.2Tl0.9Cu2Bi0.1O7', Precursors: ['SrCO3', '
   Tl2O3', 'Ti2O3', 'CuO', 'CaO', 'Bi2O3']
- Target: 'Ca3ZrSi2O9', Precursors: ['SiO2', 'CaCO3', 'ZrO2']
- Target: 'Ba3NbFe3(SiO7)2', Precursors: ['SiO2', 'Nb2O3', 'BaCO3',
   'Fe2O3']
- Target: 'Mn2Ni(CO2)6', Precursors: ['MnH6(CO)4', 'NiH6(CO)4']

Note: Ensure the quality and consistency of the example data to
   prevent parsing issues.

Generate the list of 20 precursor combinations for the target 'Na3Bi
   (AsO4)2'.
**Output Format:** Respond ONLY with a single Python-formatted list
   of lists. Each inner list should contain the precursor strings.
   Typically, 2-4 precursors per combination are expected.
Example Output Format: [['precursor1a', 'precursor1b', 'precursor1c'
   ], ['precursor2a', 'precursor2b', 'precursor2c'], , ..., ['
   precursor20a', 'precursor20b', 'precursor20c']]

**Important:** Ensure all combinations are chemically valid and
   contain all elements needed to synthesize the target material. Do
    not include any explanations or text outside the Python list
   format.
```

Listing 1: The prompt used for precursor generation is shown; all other prompts are available in our GitHub repository.
